# The apo LETM1 F‐EF‐hand adopts a closed conformation that underlies a multi‐modal sensory role in mitochondria

**DOI:** 10.1002/1873-3468.70006

**Published:** 2025-02-10

**Authors:** Qi‐Tong Lin, Danielle M. Colussi, Peter B. Stathopulos

**Affiliations:** ^1^ Department of Physiology and Pharmacology, Schulich School of Medicine and Dentistry University of Western Ontario London Canada

**Keywords:** apo, calcium sensor, F‐EF‐hand domain, LETM1, leucine zipper EF‐hand containing transmembrane protein‐1, pH sensor, solution NMR structure, temperature sensor

## Abstract

Leucine zipper EF‐hand containing transmembrane protein‐1 (LETM1) plays a critical role in mitochondrial function, with haploinsufficiency linked to Wolf‐Hirschhorn syndrome. Here, we present the solution NMR structure of the calcium (Ca^2+^)‐depleted LETM1 EF‐hand domain, revealing a closed conformation facilitated by a distinct F_1_‐helix pivot rather than decreased interhelical angle. Further, we observe regiospecific unfolding in response to hot and cold denaturation and show H662 has a pKa in‐line with physiological pH fluctuations. Finally, we demonstrate Ca^2+^‐dependent transient interactions between the EF‐hand and other LETM1 or GHITM protein domains. Collectively, our data reveal the apo‐to‐holo structural dynamics and mechanisms underlying the multi‐modal sensing by the LETM1 EF‐hand domain, highlighting its role as an adaptable regulatory element within the mitochondrial matrix.

## Graphical abstract

We present the first solution structure of the Ca^2+^‐depleted LETM1 F‐EF‐hand through a D676A/N678A Ca^2+^ binding‐deficient mutant, revealing a closed hydrophobic cleft caused by a unique F_1_‐helix pivot. The apo LETM1 F‐EF‐hand exhibits regiospecific hot and cold unfolding, sensitivity to physiological pH changes and potential for promiscuous heterotypic interactions.

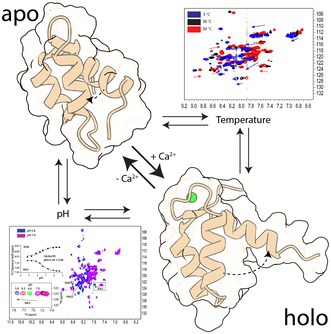

## Abbreviations

**AEA**, D676A/N678A

**apo**, Ca^2+^‐unbound

**C**, carboxyl

**CALML6**, calmodulin‐like protein 6

**CaM**, calmodulin

**CC**, coiled‐coil

**CD**, circular dichroism

**CHAPS**, 3‐[(3‐cholamidopropyl)dimethylammonio]‐2‐hydroxy‐1‐propanesulfonate

**CSP**, chemical shift perturbation

**CTD**, carboxyl‐terminal domain

**DOPE**, discrete optimized energy

**DSS**, 4,4‐dimethyl‐4‐silapentane‐1‐sulfonic acid

**EGTA**, ethylene glycol‐bis(β‐aminoethyl ether)‐N,N,N′,N′‐tetraacetic acid

**GHITM**, growth hormone inducible transmembrane

**H‐bond**, hydrogen bond

**holo**, Ca^2+^‐bound

**IMM**, inner mitochondrial membrane

**IPTG**, isopropyl β‐d‐1‐thiogalactopyranoside

**ipTM**, interface predicted template modeling score

**KEK**, D676K/N678K

**LB**, Luria broth

**LETM1**, leucine zipper EF‐hand containing transmembrane protein‐1

**MD**, molecular dynamics

**N**, amino

**N‐CaM**, calmodulin amino‐terminal domain

**NMR**, nuclear magnetic resonance

**NOE**, nuclear Overhauser effect

**NTD**, amino‐terminal domain

**PAE**, predicted aligned error

**pLDDT**, predicted local distance difference test

**pTM**, predicted template modeling score

**RBD**, ribosome binding domain

**RMSD**, root‐mean‐square deviation

**RMSF**, root‐mean‐square fluctuation

**S100A6**, calcylin

**S** ^**2**^, order parameter

**SD**, standard deviation

**SES**, D676S/N678S

**TM**, transmembrane

**VGM**, vector geometry mapping

**WHS**, Wolf‐Hirschhorn syndrome

**WT**, wild‐type

**θ**, interhelical angle

**ϕ**, pivot angle in the horizonal plane

**ω**, rotation angle

**Fig. 1:**
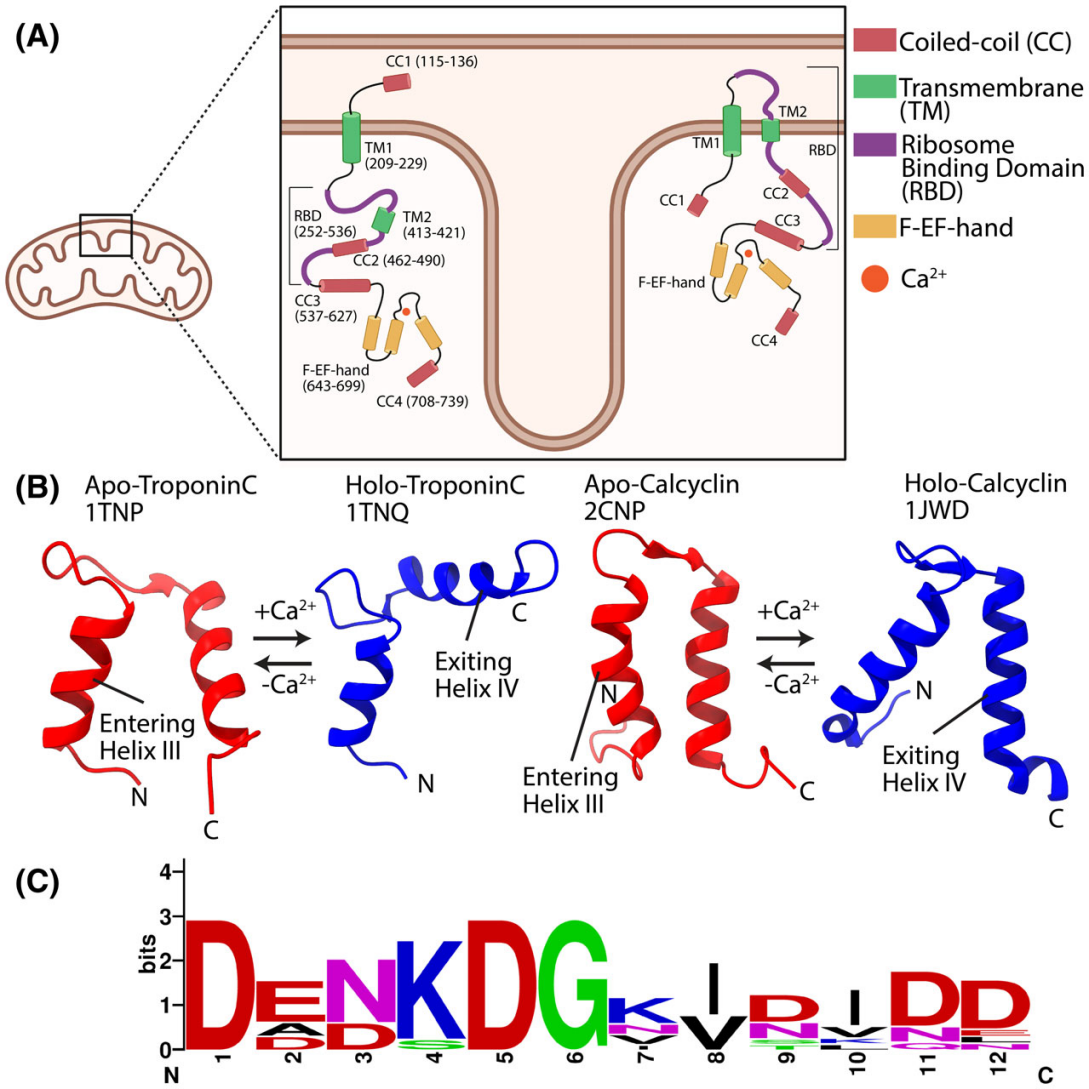
Human LETM1 proposed topologies, EF‐hand loop conservation and Ca^2+^ induced EF‐hand conformational changes. (A) Domain architecture and the topology for human LETM1 in the case of 1 × TM (left) or 2 × TM (right). The relative locations of the coiled‐coil 1, 2, 3, 4 (CC1/2/3/4, red), transmembrane 1, 2 (TM1/2, green), ribosome binding domain (RBD, magenta), and F‐EF‐hand domain (F‐EF, orange) are shown. The residue ranges are shown for each domain (left only), labeled based on UniProt annotations (O95202) and previous publications [8, 11]. (B) Apo (red) and Holo (blue) structures showing the Ca^2+^ induced conformational changes of canonical Troponin C EF2 (left) and non‐canonical calcyclin EF2 (right). (C) Consensus sequence logo plot [61] of LETM1 EF‐hand loop generated from the alignment of several LETM1 orthologues. Sequences include human (NCBI: NP_036450.1), mouse (AAH61115.1), fruit fly (NP_726453.1), roundworm (CAB03150.1), thale cress (OAP05849.1); chimpanzee (JAA40971.1), bovine (AAI20275.1), wild boar (NP_001231877.1), frog (AAI21319.1) and zebrafish (NP_001038673.1).

It has been over two decades since the Leucine zipper EF‐hand containing transmembrane protein‐1 (*LETM1*) gene was identified as one of three genes deleted in Wolf‐Hirschhorn syndrome (WHS) patients [1, 2]. Since then, a plethora of research has been conducted that describes LETM1 as a single entity with diverse impact on mitochondrial biology and cellular signaling. LETM1 is a constitutively expressed inner mitochondrial membrane (IMM) protein that has many mitochondrial roles in regulating morphology, cristae formation, protein biogenesis, bioenergetics and ion homeostasis [3, 4, 5, 6, 7, 8]. Several studies have reported the primary role of LETM1 as an antiporter exchanging protons (H^+^) for calcium (Ca^2+^) or potassium (K^+^) [5, 9, 10]. Experimental evidence suggests human LETM1 contains two transmembrane (TM) domains that orients large amino (N)‐ and carboxyl (C)‐terminal regions in the matrix [11] (Fig. 1A). The matrix‐oriented N‐terminal region contains a coiled‐coil (CC1) while the matrix‐oriented C‐terminal region contains a ribosome binding domain (RBD), three coiled‐coils (CC2‐4) and a sequence identifiable EF‐hand (Fig. 1A).

EF‐hand Ca^2+^ sensors change their conformation upon Ca^2+^ binding; moreover, the Ca^2+^ binding‐induced structural transition has been described as a ‘closed’ to ‘open’ conformational change, exposing hydrophobic residues for interactions with protein partners [12, 13, 14, 15, 16]. In archetypal Ca^2+^ sensing EF‐hands such as calmodulin (CaM) and troponin C the closed to open transition is associated with a change in the entering (*i.e*., E‐helix) and exiting (*i.e*., F‐helix) interhelical angle (θ) from ~45° (*i.e*., semi‐parallel) to ~70–90° (*i.e*., semi‐perpendicular) [17, 18] (Fig. 1B). Nevertheless, EF‐hands can diverge from this structural change. For example, Ca^2+^ binding to the N‐terminal EF‐hand domain of calcyclin (S100A6) does not induce any appreciable structural change while the C‐terminal EF‐hand domain of the same protein shows a Ca^2+^ binding‐induced open conformation [14, 19] (Fig. 1B). Thus, to fully elucidate the conformational changes that occur in a given EF‐hand, high‐resolution structures of the Ca^2+^‐free (apo) and Ca^2+^‐loaded (holo) states are needed.

Recently, we reported the structure of the holo LETM1 EF‐hand domain, revealing a unique F‐EF‐hand pairing between complete helix–loop–helix and partial loop–helix EF‐hand motifs [20]. The holo structure also showed non‐canonical Ca^2+^ coordination with absent 12th loop residue interactions (Fig. 1C) but higher solvent exposed hydrophobicity compared to holo CaM. Alphafold and similar AI programs are not yet able to accurately and consistently predict the structural consequences of ligand binding, and the LETM1 F‐EF is one such example where there are no structural changes predicted when Ca^2+^ is included in AlphaFold predictions. Functionally, we found that LETM1 F‐EF mutations that strengthen or weaken Ca^2+^ binding increase or decrease matrix Ca^2+^ levels, respectively [20], highlighting the ability of both holo and apo F‐EF conformations to regulate matrix Ca^2+^ and the apo state as an important structural target.

Here, we present a solution NMR‐derived Ca^2+^‐deplete LETM1 F‐EF‐hand domain structure, determined using the D676A/N678A Ca^2+^ binding‐deficient mutant. We find that the apo F‐EF‐hand conformation has a closed hydrophobic cleft as observed with apo CaM; however, the structural basis for the reduced solvent exposed hydrophobicity is an exiting F_1_‐helix pivot rather than closure of the E_1_ and F_1_ interhelix angle. Further, we find indication of regiospecific unfolding caused by cold and hot denaturation and a H662 pKa of ~6.58, endowing the domain with biologically significant sensitivity to pH and temperature changes. Finally, based on AlphaFold3 predictions and a prior LETM1 interaction screen [21], we examined the propensity for holo F‐EF to bind other LETM1 and growth hormone inducible transmembrane (GHITM) protein domains, observing weak interactions in both cases. Collectively, our data reveal how LETM1 F‐EF functions as a multi‐modal sensor that responds to a diverse array of environmental cues in the mitochondrial matrix.

## Materials and methods

### Plasmid constructs

Constructs encompassing human LETM1 (NCBI: NP_036450.1) residues 643–699 (F‐EF), residues 537–634 (CC3), residues 115–208 (NTD) and human GHITM (NCBI: NP_055209.2) residues 45–82 (NTD), residues 293–345 (CTD) were subcloned into pET‐28a vectors (Novagen) using NheI and XhoI restriction sites. The LETM1 F‐EF D676A/N678A mutation was previously generated using PCR‐mediated mutagenesis and confirmed by Sanger DNA sequencing of the open reading frame [20].

### Protein expression

Protein expression in BL21 (DE3) Escherichia coli cells cultured in Luria broth (LB) was induced with 400 μm isopropyl β‐d‐1‐thiogalactopyranoside (IPTG) for 4–6 h at 37 °C. Proteins were purified under denaturing conditions using nickel‐nitrilotriacetic acid agarose beads as per the manufacturer guidelines (HisPur; Thermo Scientific, Waltham, MA, USA). The lysis buffer was 30 mm Tris, 6 m guanidine‐HCl, pH 8.0, the wash buffer was 20 mm Tris, 150 mm NaCl, 6 m Urea, pH 8.0 and the elution buffer was 20 mm Tris, 150 mm NaCl, 300 mm imidazole, 6 m Urea, pH 8.0 for the metal affinity chromatography. The chaotrope was removed by dialysis in 20 mm Tris, 150 mm NaCl, pH 7.8, 4 °C using a 3500 Da molecular weight cutoff membrane (Thermo Scientific). The N‐terminal hexahistidine tag was cleaved with ~2 Units of thrombin (Sigma, St. Louis, MO, USA) per 1 mg of protein. Human LETM1 F‐EF construct final purification step was anion exchange chromatography using a QFF ANX column (Cytiva, Malborough, MA, USA) and dialysis into experimental buffers. The anion exchange chromatography was performed using 20 mm Tris, pH 7.8 and a 0–1 m NaCl gradient over 60 column volumes. In place of ANX, the Human LETM1 NTD, CTD and GHITM NTD, CTD constructs were purified with size exclusion chromatography using a Superdex 200 Increase 10/300 GL column (Cytiva) and dialysis into experimental buffers. Protein concentrations were estimated using the bicinchoninic acid assay (Pierce, Waltham, MA, USA). Experimental buffers were 20 mm Tris, 150 mm NaCl, pH 7.8 unless otherwise stated.

Uniformly ^13^C, ^15^N‐labeled protein was expressed in BL21 (DE3) *E*, *coli* cultured in M9 minimal medium with ^15^N‐NH_4_Cl (Sigma) and D‐Glucose‐^13^C (Cambridge Isotope Laboratories, Tewksbury, MA, USA) as the sole nitrogen and carbon source, respectively. Purification was performed as per the LB‐expressed protein. NMR buffers were 20 mm Tris pH 7.8, 50 mm NaCl, 10 mm 3‐[(3‐cholamidopropyl)dimethylammonio]‐2‐hydroxy‐1‐propanesulfonate (CHAPS) with 30 mm CaCl_2_ (Holo) and without 30 mm CaCl_2_ (Apo). Sixty μm 4,4‐dimethyl‐4‐silapentane‐1‐sulfonic acid (DSS) and 10% (v/v) D_2_O were added to all NMR samples for referencing.

### Solution NMR spectroscopy

NMR experiments were performed on a Bruker Avance NEO 600 MHz spectrometer equipped with a TXI S3 ^2^H, ^1^H, ^15^N, ^13^C room temperature probe, at 35 °C unless otherwise stated (Schulich Biomolecular NMR Facility, Western University, London, ON, Canada). Backbone atom chemical shift assignments were obtained from ^1^H‐^15^N‐HSQC, HNCO, CBCA(CO)NH and HNCACB experiments. Side chain assignments were from ^1^H‐^13^C‐HSQC, (H)C(CO)NH‐TOCSY, H(C)(CO)NH‐TOCSY and HCCH‐TOCSY experiments. NOE peaks were from ^15^N‐edited and ^13^C‐edited NOESY‐HSQC experiments. All NMR data transformation and processing were done using NMRPipe v10.9 [22]; assignments, peak position, and intensity analyses were performed using XEASY/NEASY [23].

### Structural determination and analysis

The dihedral and hydrogen bond restraints were derived from the assigned chemical shifts for apo LETM1 F‐EF D676A/N678A using TALOS‐N [24]. These restraints added to CYANA (v2.1) [25, 26], used for simultaneous NOE assignment, picked from ^15^N‐ and ^13^C‐edited NOESY spectra, and structural ensemble calculation. The resultant CYANA‐generated structure was water‐refined in CNS (v1.3) [27, 28, 29], including dihedral, hydrogen bond and CYANA‐assigned NOE/distance restraints. Structural homologs were identified using the DALI server [29], and the extraction of the helical angles was performed using Vector Geometry Mapping (VGM) [30]. All other structural analyses and images were performed and rendered using ChimeraX (v1.8.1) [31].

The NMR sample pH titration was performed by adding increments of 0.5–1.5 μL of 1 m HCl to reach the desired pH, where volume additions were empirically determined. The sample pH was measured using the chemical shifts for the protonated and deprotonated forms of the internal pH indicators formate and imidazole included in the sample [32]. The ^1^H chemical shifts of select residues were plotted over the pH range and fitted to a modified Henderson‐Hasselbalch equation

$$\delta_{\mathrm{obs}}=\left(\left(10^{\mathrm{pH}-\mathrm{pKa}}\right)\delta_{A}+\delta_{\mathrm{HA}}\right){\left(1+10^{\mathrm{pH}-\mathrm{pKa}}\right)}^{-1}.$$

For interaction analyses, NMR samples of uniformly ^15^N‐labeled LETM1 F‐EF WT were mixed with human LETM1‐CC3, LETM1‐NTD, GHITM‐NTD or GHITM‐CTD protein constructs in a 1 : 2 to 1 : 3 molar ratio. All constructs were prepared by dialysis in NMR buffer with 30 mm CaCl_2_.

### Homology modeling and MD simulations

Homology models of WT, D676K/N678K (KEK) and D676S/N678S (SES) LETM1 F‐EF (*i.e*., human LETM1 residues 643–699) were generated using the lowest energy, solution NMR D676A/N678A (AEA) LETM1 F‐EF structure (9DMR) as a template in modeler v10.4 [33]. For each WT and mutant protein, 10 homology models were generated using the default settings, and the models with the lowest discrete optimized protein energy (DOPE) score were taken for further analyses. MD simulations were conducted using GROMACS 2022.3 [34] and the OPLS‐AA/L all‐atom force field [35]. The simple point charge SPC216 water model was used to solvate the systems in a cubic box with a solute‐box distance of 1.0 nm. Simulations were done with neutral protein termini, and water was replaced with Na^+^ ions to neutralize other net charges. The solvated, electroneutral systems were then relaxed through steepest descent energy minimization. Systems were equilibrated for the NVT ensemble (*i.e*., fixed number of atoms, volume, and temperature) at a constant temperature of 310 K (*i.e*., ~37 °C) for 100 ps using the modified Berendsen's thermostat algorithm [36]. The second equilibration phase was under NPT ensemble (*i.e*., fixed number of atoms, pressure, and temperature) for 100 ps using the Parrinello‐Rahman barostat pressure [37] of 1 bar. The final MD simulations were conducted for 500 ns under NPT ensemble conditions.

Root‐mean‐square deviation (RMSD)s were calculated for the Cα atoms of residues 647–695, (*i.e*., excluding the flexible N‐ and C‐terminal regions, only processing every 10^th^ frame). Root‐mean‐square fluctuations (RMSF) of Cα were also calculated for all residues over the 500 ns. The rotation and translation of proteins were removed from the final trajectory files to calculate S^2^ order parameters, excluding the T643 (N‐terminal) and P664 residues. The autocorrelation functions were generated using a 2nd order Legendre polynomial before calculating the residue‐specific S^2^ order parameters with a perl script. To visualize the 500 ns trajectory files as movies, periodic boundary conditions were set (−pbc nojump and ‐pbc mol), and the rotational and translational motions were removed (−fit rot+trans), processing every 10th frame. RMSD, RMSF and S^2^ were calculated using GROMACS 2022.3 tools [34], the movies were generated in ChimeraX [38] and snapshot images of the proteins at 0 ns and 500 ns were generated using PyMoL 2.5.2 (Schrödinger LLC, New York, NY, USA).

### Far‐UV CD spectroscopy

Thermal melts were recorded using a 1 mm pathlength quartz cuvette by monitoring the change in CD signal at 222 nm from 20 to 95 °C. A scan rate of 1 °C min^−1^, 1 nm bandwidth and 8 s averaging time was used for these thermal measurements, recorded on a Peltier temperature‐controlled Jasco J‐810 spectrometer (Jasco, Inc., Easton, MD, USA).

## Results

### Apo D676A/N678A LETM1 EF1 (residues 643–699) is Ca^2+^‐insensitive and maintains a unique F‐EF‐hand pairing

**Fig. 2:**
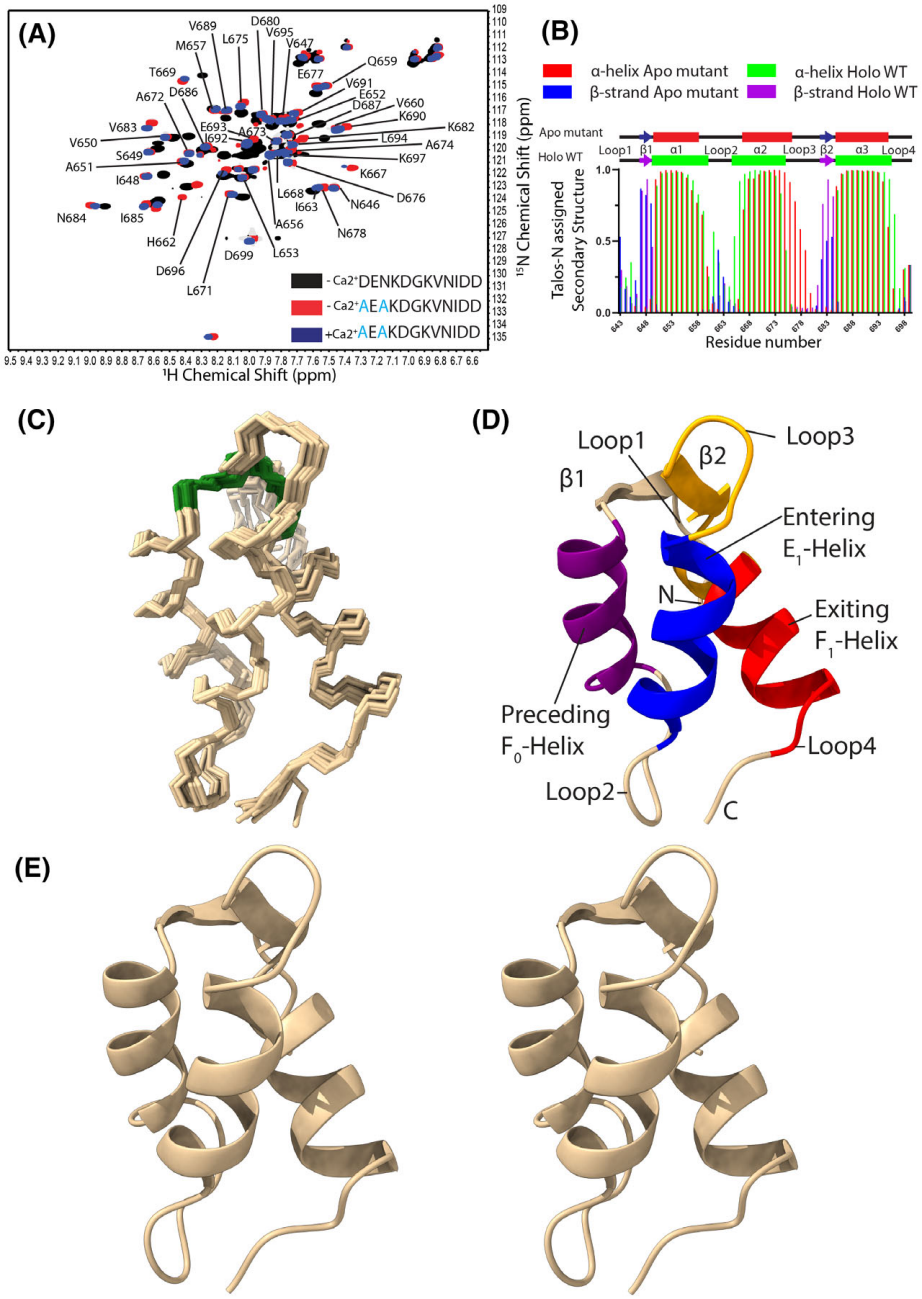
Solution structure of the apo D676A/N678A LETM1 F‐EF‐hand domain. (A) Assigned ^1^H‐^15^N HSQC spectrum of D676A/N678A LETM1 F‐EF acquired in the absence of Ca^2+^ (red), overlaid with WT LETM1 F‐EF in the absence of Ca^2+^ (black) and LETM1 F‐EF D676A/N678A in the presence of 30 mm CaCl_2_. Assigned amide crosspeaks are labeled. (B) TALOS‐N derived secondary structure of LETM1 F‐EF based on chemical shift assignments [24]. The secondary structure elements are shown at top relative to the LETM1 residue number (apo: α‐helix, red cylinder; β‐strand, blue arrow; holo: α‐helix, green cylinder; β‐strand, purple arrow). (C) Superposition of the 10 lowest energy structures showing mainchain bond connectivity. The small β‐sheet formed by H‐bonding between the partial loop1‐F_0_‐helix and canonical E_1_‐helix–loop3‐F_1_‐helix is shaded green. (D) Cartoon representation of the lowest energy LETM1 F‐EF structure highlighting the relative locations of the preceding F_0_‐helix (α1; purple), entering E_1_‐helix (α2; blue), exiting F_1_‐helix (α3; red), Ca^2+^ binding loop (orange). (E) Stereo view of the lowest energy structure in cartoon representation.

^1^H‐^15^N HSQC spectra of uniformly ^15^N‐labeled wild‐type (WT) EF1 (residues 643–699; named F‐EF [20]) in the Ca^2+^ depleted state showed only ~77% of the expected ^1^H‐^15^N (amide) cross‐peaks and poor dispersion within the central region of the spectrum (Fig. 2A). However, the D676A/N678A F‐EF double mutant we previously characterized as Ca^2+^ binding‐deficient [20] showed ~89% of the expected amide cross‐peaks, improved dispersion and increased homogeneity of peak intensities. Thus, we used the D676A/N678A LETM1 F‐EF double mutant as a surrogate for the WT apo LETM1 F‐EF structure. With the high‐quality spectra, we assigned ~85% of the ^1^H, ^15^N, ^13^C resonances in the Ca^2+^ deplete condition. As expected, the G681 amide ^1^H signal in the Ca^2+^ binding loop was not downfield shifted in either the presence of high (*i.e*., 30 mm CaCl_2_) or absence of Ca^2+^, consistent with a Ca^2+^ binding‐deficient and Ca^2+^‐insensitive apo conformation (Fig. 2A). The secondary structure of D676A/N678A F‐EF assigned from the chemical shifts using TALOS‐N [24] revealed the apo structure is comprised of three α‐helices and two short β‐strands (Fig. 2B).

To elucidate the solution structure of apo D676A/N678A F‐EF we included chemical shift/TALOS‐N [24] derived torsion angle and hydrogen (H)‐bond restraints in CYANA [25] during assignment of 1533 nuclear Overhauser effect (NOE) peaks from a total of 2171 picked from ^13^C‐ and ^15^N‐edited NOESY experiments. The resultant CYANA structure was water‐refined [27] to produce a 10 lowest energy structural ensemble well defined by the NMR constraints (Table 1). The all‐heavy atom root‐mean‐square deviation (RMSD) of the ensemble was 1.02 ± 0.11 Å with no NMR‐derived distance or dihedral violations beyond 0.2 Å or 6.6 °, respectively, as well as 88.5% of the torsion angles occupying the most favorable Ramachandran regions and 99.6% overall in the allowed regions (Fig. S1). The structure is made up of a loop1 (T643‐N646), β1‐strand (V647‐S649), α1‐helix (V650‐K658; F_0_), loop2 (Q659‐S666), entering α2‐helix (K667‐A676; E_1_), loop3 (E677‐G681), β2‐strand (K682‐N684), exiting α3‐helix (I685‐V695; F_1_) and loop4 (D696‐D699) (Fig. 2B,C). Similar to holo LETM1 F‐EF (9BA1 [20]) and most EF‐hand pairs in nature [12, 14], the apo F‐EF mutant maintains a short, antiparallel β‐sheet through backbone H‐bonds from I648 and V683, pairing loop1 with loop3 (Fig. 2D,E). Intriguingly, the apo conformation shows shorter α‐helices, where the preceding F_0_ helix (α1) is reduced by two residues (*i.e*., holo α1 = V650‐V660; apo α1 = V650‐K658), entering E_1_ helix (α2) is reduced by one residue (*i.e*., holo α2 = E665‐L675; apo α2 = K667‐A676) and exiting F_1_ helix (α3) is shortened by one residue (*i.e*., holo α3 = I685‐D696; apo α3 = I685‐V695) compared to holo (Fig. 2B).

**Table 1:** Solution NMR structure statistics summary. SD, standard deviation.

Distance and dihedral restraints	
Total NOE distance limits	859
Intra‐residue	152
Sequential: $\left|i-j\right|=1$	216
Medium range: $1<\left|i-j\right|<5$	225
Long‐range: $\left|i-j\right|\geq 5$	266
Hydrogen bonds^a^	
α‐helix	21 × 2
β‐strand	2 × 3
Total dihedral angle restraints^a^	
ϕ	46
ψ	46
Violations (mean ± SD)^b^	Global (residues 643–699)
Number of distance violations >0.2 Å	0
Number of dihedral violations >5°	0.70 ± 0.67
Maximum distance constraint violation (Å)	0.17 ± 0.02
Maximum dihedral angle violation (°)	5.29 ± 0.84
Distance constraint RMSD (Å)	0.02 ± 0.00
Dihedral constraint RMSD (°)	1.13 ± 0.11
Idealized geometry deviations (mean ± SD)^b^, ^c^	all residues
Bond length RMSD (Å)	0.01 ± 0.00
Bond angle RMSD (°)	1.44 ± 0.03
Improper RMSD (°)	1.73 ± 0.07
Ramachandran statistics (% of residues)^d^	
Most favorable regions	88.5
Allowed regions	11.1
Generously allowed regions	0.0
Disallowed regions	0.4
Average pairwise RMSD (Å)^e^	
Heavy atom (to mean)	1.02 ± 0.11
Backbone atom (to mean)	0.53 ± 0.14

^a^ Determined from all‐atom chemical shifts using TALOS‐N

^b^ Calculated for the 10 lowest energy structures using RECOORD in CNS.

^c^ Calculated including N‐terminal cloning artifact (GSHMAS).

^d^ Calculated using PROCHECK‐NMR

^e^ Calculated in PyMOL relative to the closest to mean model 5.

Overall, the apo mutant structure maintains the unique pairing between partial (loop1‐F_0_‐helix) and full (E_1_‐helix‐loop3‐F_1_‐helix) EF‐hand motifs previously elucidated for the holo state [20] but with reduced levels of α‐helicity.

### Apo D676A/N678A is less dynamic than the WT LETM1 F‐EF‐hand protein

We next acquired ^1^H‐^15^N HSQC spectra in the absence and presence of 5 mm ethylene glycol‐bis(β‐aminoethyl ether)‐N,N,N′,N′‐tetraacetic acid (EGTA) to confirm that residual Ca^2+^ is not bound to D676A/N678A F‐EF. Most ^1^H(^15^N) amide peaks were not altered by the Ca^2+^ chelator addition (*i.e*., ~86%), indicating a largely unchanging structure; however, a loss of H662, K667 and T669 peak intensities was apparent upon the EGTA addition (Fig. S2A). These residues are localized to F_0_‐E_1_ connecting loop2, which is an electrostatically positive surface region of D676A/N678A (Fig. S2B,C). Thus, these minor perturbations are likely caused by transient interactions between electronegative EGTA and the positively charged area of the loop2 region. We also used AlphaFold3 to probe the possibility that a second Ca^2+^ binding site exists by predicting the F‐EF structure in the presence of two Ca^2+^ ions. While one Ca^2+^ ion was confidently predicted within the canonical EF1 binding loop, consistent with our previous holo WT F‐EF structure [39], the second Ca^2+^ ion was placed at an interaction site on coiled‐coil 3 with low confidence (Fig. S2D).

Next, we probed the potential effect of the mutations on the F‐EF structure and dynamics. First, we created WT, D676K/N678K (KEK) and D676S/N678S (SES) homology models using the experimentally determined D676A/N678A (AEA) structure as the template [33], finding all structures were highly similar with backbone RMSDs of 0.11, 0.12, and 0.13 Å for WT versus AEA, KEK versus AEA and SES versus AEA, respectively. The SES structure showed slightly improved Ramachandran statistics (*i.e*., 92% and 8% of dihedral angles in the most favored and allowed regions, respectively) compared to the WT, KEK, and AEA structures (*i.e*., 89% and 11% in the most favored and allowed regions, respectively). Subsequently, we performed all‐atom molecular dynamics (MD) in explicit water simulations. Over the 500 ns simulation time, all four proteins maintained analogous three‐helix packing and comparable starting versus endpoint backbone structural changes of 3.2, 3.7, 3.8, 2.6 Å for AEA, WT, KEK, and SES, respectively (Fig. S3A–E). To evaluate dynamics, we calculated root‐mean‐squared fluctuation (RMSF) and the S^2^ order parameter on a per residue basis over the 500 ns. The AEA structure exhibited the highest N‐H bond vector rigidity on the ps‐ns timescale (S^2^) across the F_0_, E_1_, and F_1_ helices compared to the WT, SES, and KEK models (Fig. S3F). Congruently, the Cα RMSF for AEA was lowest across the F_0_, E_1_ and F_1_ helices compared to the WT, SES, and KEK models (Fig. S3G).

Overall, these data confirm that LETM1 F‐EF binds a single Ca^2+^ ion, which is abrogated upon introducing the D676A/N678A (AEA) mutation [20]. Moreover, while different side chains can likely be accommodated structurally at the 676 and 678 positions on a short timescale, the AEA mutation decreases the dynamics of the F‐EF secondary structure components. The increased dynamics of WT and KEK compared to AEA, probably underlies the poorer quality ^1^H‐^15^N‐HSQC spectra [20] and solution structure intractability for those proteins.

### Apo LETM1 F‐EF adopts a closed conformation while maintaining semi‐perpendicular interhelical angles

**Fig. 3:**
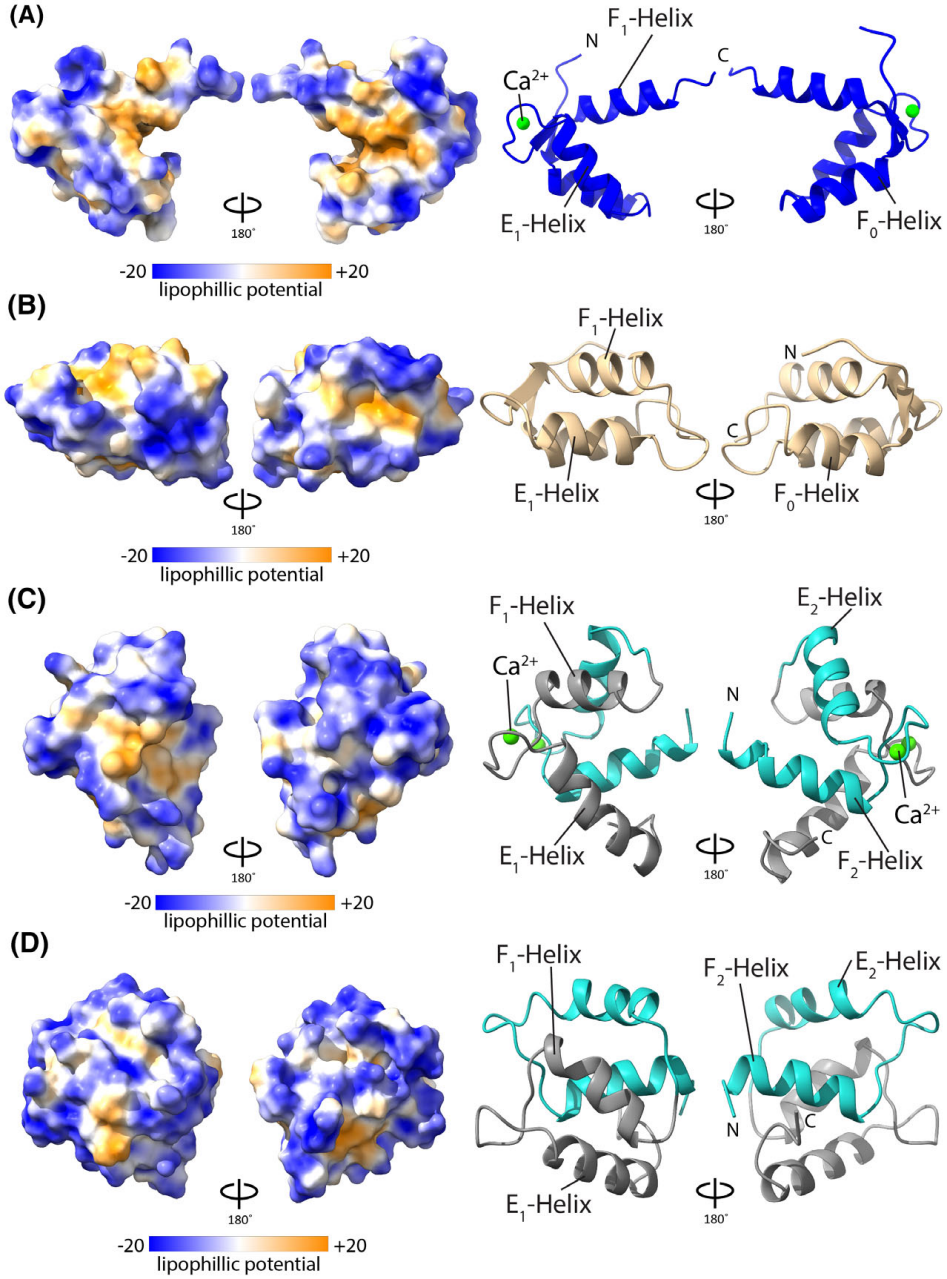
Lipophilicity potential maps of the apo and holo human LETM1 F‐EF compared to N‐terminal CaM. (A) Lipophilicity potential plotted on the surface of the holo WT human LETM1 F‐EF (left) and the associated backbone conformation as cartoon representation (right; blue). (B) Lipophilicity potential plotted on the surface of the apo D676A/N678A human LETM1 F‐EF (left) and the associated backbone conformation as cartoon representation (right; beige). (C) Lipophilicity potential plotted on the surface of the holo N‐terminal CaM (left) and the associated backbone conformation as cartoon representation (right; EF1, gray; EF2, cyan). (D) Lipophilicity potential plotted on the surface of the apo N‐terminal CaM (left) and the associated backbone conformation as cartoon representation (right, EF1, gray; EF2, cyan). In A–D, lipophilicity is colored from +20 to −20 *K*_ow_·Å using an orange (hydrophobic) to blue (hydrophilic) gradient, respectively. Ca^2+^ ions are shown as green spheres.

Ca^2+^ sensing EF‐hands typically undergo a structural change upon binding Ca^2+^, resulting in exposure of hydrophobic groups for interactions with binding partners [40]. Previously we discovered that holo LETM1 F‐EF, despite lacking two complete EF‐hand motifs, contains 32% more solvent accessible hydrophobic surface area compared to N‐terminal CaM (N‐CaM), the closest structural homolog in the Ca^2+^‐loaded state [20]. Here, we find that the apo F‐EF mutant adopts a closed conformation that reduces the solvent exposed hydrophobic surface area by 24% (Fig. 3A,B, Table 2). Comparatively, apo N‐CaM shows ~9% less solvent accessible hydrophobic surface area compared to holo N‐CaM (Fig. 3C, D, Table 2). Remarkably, apo LETM1 F‐EF contains ~16% more solvent accessible hydrophobic surface area compared to holo N‐CaM, suggesting a potential for interactions with binding partners in both the presence and absence of Ca^2+^ (Table 2).

**Table 2:** Summary of Hydrophobic SASA and vector geometry mapping.

	LETM1 F‐EF		CaM NTD	
Ca^2+^	+	−	+	−
Hydrophobic SASA (Å^2^)^a^	1341	1080	1010	927
θ (°)^b^	68	69	62	45
ϕ (°)^c^	87	164	89	109
ω (°)^d^	237	22	280	300
Outer distance (Å)^e^	16.8	11.1	16.3	13.2
Midpoint distance (Å)^f^	12.4	8.8	12.2	12.0
Inner distance (Å)^g^	10.1	11.1	10.5	12.6

^a^ Solvent‐accessible surface are calculation for residues Ala, Val, Leu, Ile, Pro, Phe, Met and Trp in ChimeraX v.1.8.1.

^b^ Angle θ between the entering and exiting helices.

^c^ Angle ϕ the horizontal plane angle measured between the +x axis and the xy projection of the exiting helix.

^d^ Angle ω the counterclockwise angle of rotation about the exiting helix vector axis.

^e^ Outer distance defined by the interhelical distance between the N‐terminal end of the entering helix and the C‐terminal end of the exiting helix.

^f^ Midpoint distance defined by the interhelical distance between the midpoints of the entering and exiting helix vectors.

^g^ Inner distance defined by the interhelical distance between the C‐terminal end of the entering helix and N‐terminal of the exiting helix.

**Fig. 4:**
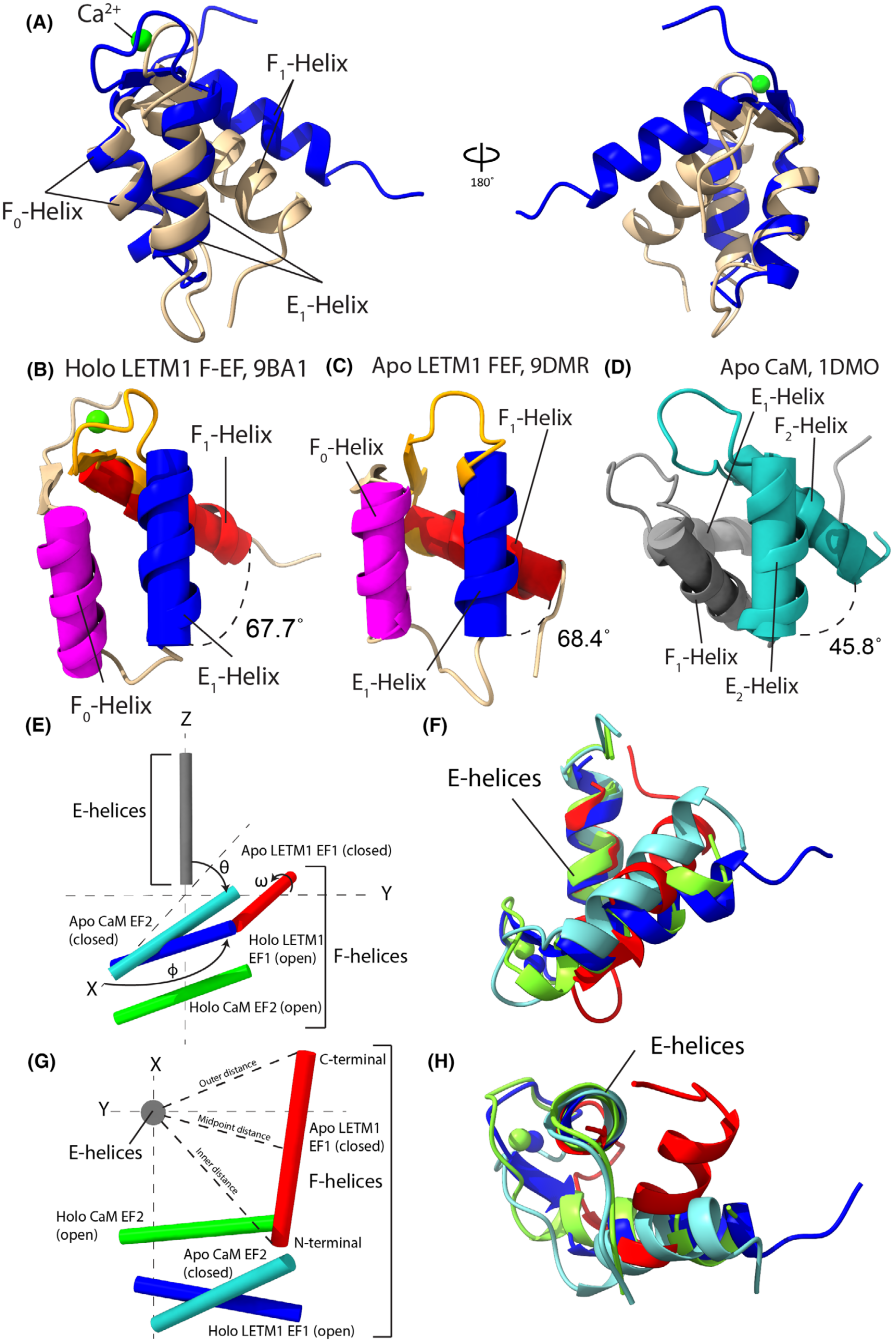
Structure comparison of apo D676A/N678A LETM1 EF1, holo WT LETM1 and apo CaM. (A) Backbone Cα structural alignment of the apo D676A/N678A LETM1 F‐EF (beige; 9DMR) and holo WT LETM1 F‐EF (blue; 9BA1). The all‐atom RMSD was 6.4 Å between 57 matched residues (*i.e*., 643–699) and 2.3 Å when aligning only preceding F_0_ and entering E_1_ helices, calculated in ChimeraX. (B) Holo LETM1 F‐EF interhelical angle between the entering E_1_ (blue) and exiting F_1_ (red) helices. (C) Apo D676A/N678A LETM1 F‐EF interhelical angle between the entering E_1_ (blue) and exiting F_1_ (red) helices. (D) Apo N‐terminal CaM (EF1, gray; EF2, cyan) interhelical angle in the closed/semi‐parallel conformation. (E) Vector geometry mapping (VGM) [30] of the apo mutant and holo WT LETM1 EF1 compared to apo and holo N‐terminal CaM EF2. The entering helices of N‐terminal CaM EF2 and LETM1 EF1 were aligned with the entering helix of the apo N‐terminal CaM EF2 (set as the reference) to highlight the relative interhelical θ angles of the exiting helices. The positions of the last 11 backbone N, Cα and C(O) atoms in the entering and exiting helices were averaged to generate the vectors. (F) Backbone Cα structural alignment of apo mutant and holo WT LETM1 EF1 compared to apo and holo N‐terminal CaM, oriented as per panel E to highlight the interhelical θ angles. (G) Top‐down VGM view of apo mutant and holo WT LETM1 EF1 compared to apo and holo N‐terminal CaM to highlight the exiting pivot ϕ angles. (H) Backbone Cα structural alignment of apo mutant and holo WT LETM1 EF1 compared to apo and holo N‐terminal CaM, oriented as per panel G to highlight the pivot ϕ angles. In E–H, apo D676A/N678A LETM1 EF1 is red, holo WT LETM1 EF1 is blue, apo N‐terminal CaM EF2 is cyan and holo N‐terminal CaM EF2 is green. In E and G, entering helices are gray.

Structural alignment of holo and apo LETM1 F‐EF reveals large conformational changes (*i.e*., all‐atom RMSD of 5.3 Å), highlighted by a pivoting of the exiting F_1_‐helix and reorientation of the Ca^2+^ binding loop (Fig. 4A). Specifically, the exiting F_1_‐helix extends away from the F_0_/E_1_‐helix pair in the holo state while packing tightly against these helices in the apo state. Further, the Ca^2+^ binding loop (loop3) is pulled forward as the F_1_‐helix pivots to interact with F_0_/E_1_ helices in the apo state (Fig. 4A). The preceding F_0_ and entering E_1_ helices show very little change between holo and apo states with an all‐atom RMSD of 1.0 Å between the two structures.

We used vector geometry mapping [30] to objectively quantify the interhelical angle (θ), pivot angle in the horizonal plane (ϕ) and rotation angle (ω) of the E_1_ relative to F_1_ helices making up the Ca^2+^‐binding EF‐hand. The LETM1 E_1_‐F_1_‐interhelical angle, θ, was found to be ~68° in the Ca^2+^ − loaded state, similar to the open state of holo N‐CaM EF2 (Fig. 4B, Table 2). Surprisingly, we found the E_1_‐F_1_‐interhelical angle did not change from this semi‐perpendicular relative orientation in the apo LETM1 F‐EF mutant structure (*i.e*., θ ~68°), in stark contrast to the semi‐parallel orientation adopted by apo N‐CaM EF2 (*i.e*., θ ~46 °) (Fig. 4C,D, Table 2). Instead, when the E_1_ helix is aligned along the Z‐axis, the apo LETM1 F_1_ helix shows a ~77° greater counterclockwise pivot in the horizontal X‐Y plane in the apo versus holo state (*i.e*., ϕ ~164° versus 87°, respectively); further, apo F‐EF shows a decreased twist angle, ω, from 237° in the holo state to 22° in the apo state (Fig. 4E,F, Table 2). These angular changes culminate in much closer packing between the E_1_ and F_1_ helices in the apo state, as evidenced by decreased E_1_‐F_1_ midpoint and E_1_‐F_1_ outer helix distances by 3.6 and 5.7 Å, respectively, in the apo F‐EF state compared to the holo state. In contrast, the midpoint and outer helix distances decrease by only 0.2 and 3.1 Å, respectively, in N‐CaM in the apo versus holo states (Fig. 4G,H, Table 2).

Taken together, we find that the LETM1 F‐EF‐hand adopts a conformation where closure of the hydrophobic cleft is not due to a more acute E_1_‐F_1_ interhelical angle (θ), but rather an increase in horizontal plane angle (ϕ), bringing the exiting F_1_‐helix closer to the E_1_‐helix.

### Apo LETM1 F‐EF most closely resembles the DNA binding protein RuvA but is predicted to be structurally homologous to EF‐hand‐containing CALML6

**Fig. 5:**
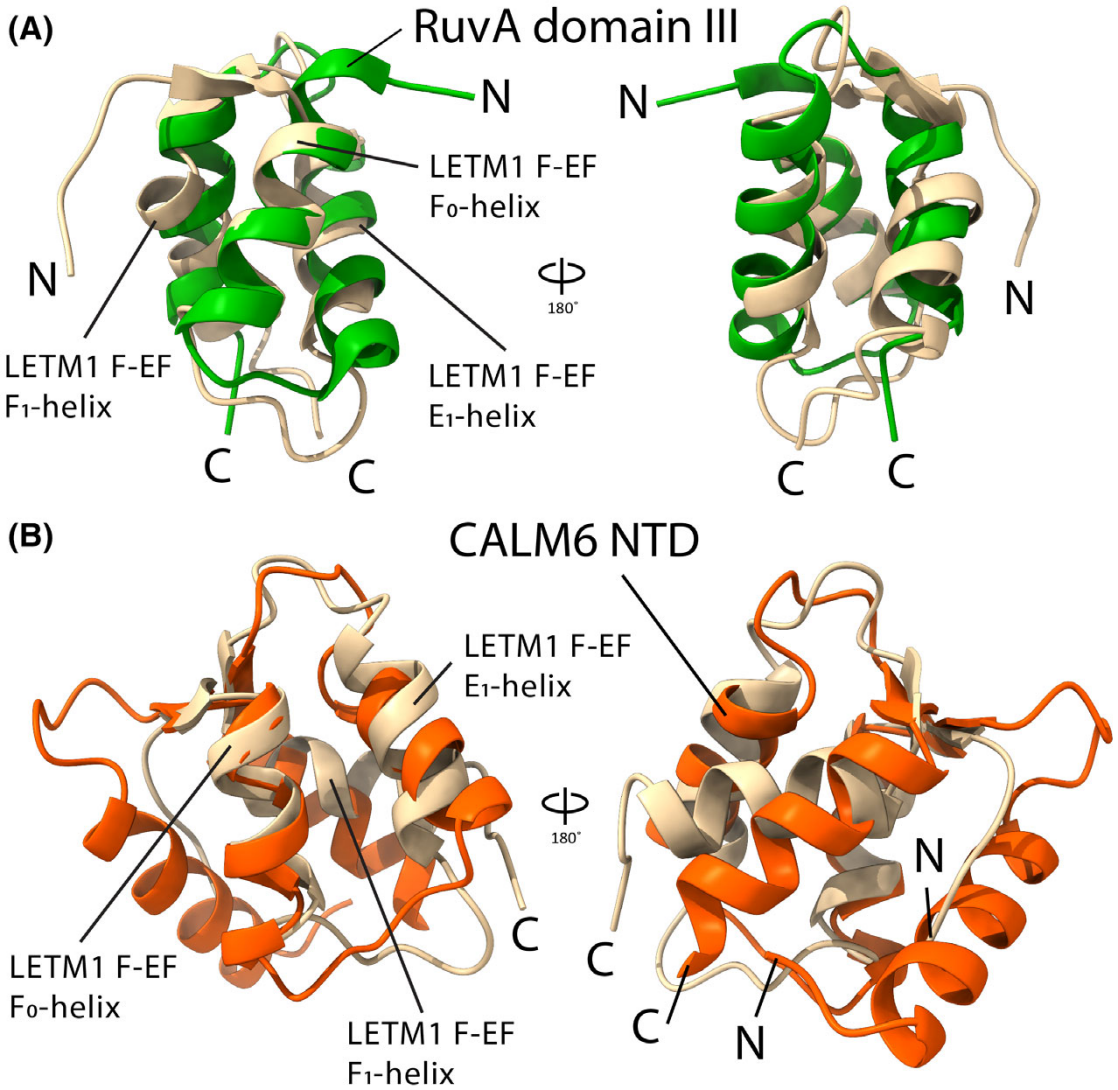
Structural alignment the apo D676A/N678A LETM1 F‐EF with RuvA and CALML6. (A) Backbone Cα structural alignment of the apo D676A/N678A LETM1 F‐EF (beige; 9DMR) and RuvA domain III (green; 1IXS). The Cα and all‐atom RMSDs are 2.2 Å and 5.2 Å, respectively, between 45 matched residues (*i.e*., 651–697 in LETM1 and 148–200 in RuvA), calculated in ChimeraX. (B) Backbone Cα structural alignment of the apo D676A/N678A LETM1 F‐EF domain (beige; 9DMR) and CALML6 (orange; UniProt: Q8TD86). The Cα and all‐atom RMSDs are 3.1 and 5.1 Å, respectively, between 54 matched residues (*i.e*., 643–699 in LETM1 and 48–105 in CALML6), calculated in ChimeraX. In A and B, cartoon representations of the backbone conformations are shown and the amino (N) and carboxyl (C) termini are labeled.

To gain more insight into potential physiological roles of the domain, we searched for structural homologs of apo LETM1 F‐EF among known experimentally determined structures in the Protein Data Bank using DALI [29]. Surprisingly, we found the most commonly identified structural homolog to be the carboxyl‐terminal domain (CTD) of RuvA from *Mycobacterium leprae* (UniProt: P40832) with a Cα RMSD of 2.2 Å between 45 matched residues (*i.e*., within 651–697 of LETM1 and 148–200 of RuvA) (Fig. 5A). RuvA specifically recognizes and binds to Holliday junctions that form during homologous recombination and DNA repair [41] as a protein–protein interaction domain (see Discussion). Additionally, we queried for structure homologs among the predicted human protein structures in AlphaFold Protein Structure Database [42]. Intriguingly, calmodulin‐like protein 6 (CALML6; UniProt: Q8TD86) was among the top predicted human structural homologs of apo LETM1 F‐EF. CALML6, which has ~45% sequence identity and ~75% sequence similarity to CaM, shows a Cα RMSD with apo LETM1 F‐EF of 3.1 Å between 53 matched residues (*i.e*., within 643–697 of LETM1 and 43–102 of CALML6). CALML6 contains four putative EF‐hand motifs making two EF‐hand domains linked through an extended α‐helix. The N‐domain of CALML6 is well‐predicted by AlphaFold (*i.e*., pLDDT >0.7 for all residues that structural align with apo LETM1 F‐EF (Fig. 5B)). Thus, despite apo LETM1 F‐EF adopting a peculiar ϕ angle to reduce solvent exposed hydrophobicity rather than a semi‐parallel θ angle as observed with CaM, other CaM‐like proteins such as CALML6 are predicted to be structurally similar, reinforcing a role for LETM1 F‐EF as a sensory domain. Further, the homology with RuvA may imply that, even in the apo state, F‐EF may mediate heterotypic protein–protein interactions, consistent with the high solvent exposed hydrophobic surface area, on par with the holo N‐CaM (Table 2).

### LETM1 F‐EF H662 has a more basic pKa in the apo versus holo state

Centrally positioned in loop2 between the preceding F_0_ and E_1_‐helices, H662 is the principal residue responsible for the structural sensitivity of apo F‐EF to pH changes in the 6.0–8.0 range [20]. We previously evaluated the protonation state, tautomer equilibrium and pKa of the H662 sidechain in the WT LETM1 F‐EF‐hand domain and found a pKa of ~6.28 and ~6.39 in the holo and apo states, respectively [20]. We also found that H662, located in loop2, undergoes significant chemical shift perturbations (CSPs) when the last two residues in the Ca^2+^ binding loop (loop3) are mutated [20]. Hence, H662 is sensitive to structural alterations in the distant loop3, likely transmitted through the E_1_‐helix. The DelPhipKa webserver [43] predicts a pKa of 6.32 for H662 based on the apo LETM1 F‐EF mutant structure, less alkaline than the 6.71 prediction for the holo WT F‐EF structure (9BA1), suggesting a conformation‐dependent pKa for H662 of LETM1 F‐EF.

**Fig. 6:**
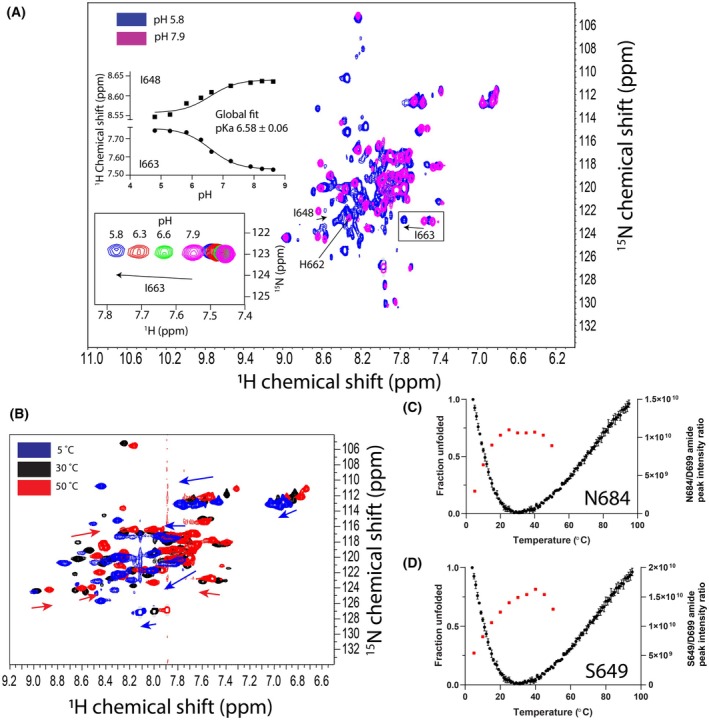
pH and temperature‐dependent chemical shift perturbations. (A) Overlaid ^1^H‐^15^N HSQC spectra of apo D676A/N678A LETM1 F‐EF at pH 7.9 (magenta) and 5.8 (blue). Top inset shows the ^1^H chemical shifts of the I663 (squares) and I648 (circles) amides plotted as a function of pH and globally fitted to a modified Henderson‐Hasselbalch equation (solid lines; see methods), revealing a pKa of 6.58 ± 0.06. Bottom inset shows the overlaid I663 amide crosspeaks at all pH values evaluated. Residues H662, I663 and I648 are labeled. Imidazole and formate chemical shifts included in the buffers were used to precisely assign pH of each solution. (B) Overlaid ^1^H‐^15^N HSQC spectra of apo D676A/N678A LETM1 F‐EF acquired at 5 (blue), 30 (black) and 50 °C (red). Red and blue arrows highlight regiospecific CSP directions induced by cold (5 °C) and hot (50 °C) temperatures, respectively, compared to the 30 °C spectrum. (C) Changes in normalized N684 amide peak intensity (red squares) from ^1^H‐^15^N‐HSQC spectra and far‐UV CD signal at 222 nm (black circles) relative to temperature. (D) Changes in normalized S649 amide peak intensity (red squares) from ^1^H‐^15^N‐HSQC spectra and far‐UV CD signal at 222 nm (black circles) relative to temperature. In C and D, temperatures favoring the most folded conformation in the CD data coincided with most intense amides in the NMR spectra, and the same CD thermal melt data represented as mean ± SD from *n* = 3 separate experiments are shown.

To experimentally confirm the predicted pKa value for D676A/N678A apo LETM1 F‐EF, we acquired a series of ^1^H‐^15^N HSQC spectra at pH 5.0–9.0. The largest CSPs were observed in the pH 6.0–8.0 range, where a more acidic pH led to an increased number of visible amide crosspeaks but also more overlap between peaks (Fig. 6A). The protonation state of the imidazole is highly affected by its local environment. Thus, CSPs of nuclei close to ionizable moieties can provide a reliable measure of the pKa [44]. In our titration, while the H662 amide was not well resolved in the acidic pH regime, the immediately adjacent I663 and I648 amide chemical shifts were easily tracked throughout the titration. Globally fitting the ^1^H(^15^N) CSPs observed for I663 and I648 as a function of pH to a modified Henderson‐Hasselbalch equation revealed a H662 pKa of 6.58 ± 0.06 for the apo LETM1 F‐EF mutant (Fig. 6A). Therefore, the D676A/N678A apo F‐EF mutant shows a slightly more basic pKa compared to WT apo F‐EF, but both values are within the physiological range of pH experienced in the matrix [45].

### Disparate apo LETM1 F‐EF regions undergo unfolding at cold and hot temperatures

Apo LETM1 F‐EF displays cold unfolding at temperatures below ~37 °C and heat denaturation at temperatures above ~42 °C [46]. Ca^2+^ binding to LETM1 F‐EF increases the stability of the domain, abrogating the cold unfolding observed at near physiological temperature (*i.e*., ~37 °C) [46]. To ascertain the effects of cold and hot temperatures on the F‐EF structure, we acquired a series of ^1^H‐^15^N‐HSQCs using the Ca^2+^‐binding‐deficient apo LETM1 F‐EF mutant between 5 and 50 °C. Overlaid ^1^H‐^15^N‐HSQC spectra acquired at 5, 30, and 50 °C highlight the collapse of crosspeaks toward the central ~8.0–8.5 ppm region for the 5 and 50 °C spectra compared to the 30 °C spectrum, where the protein is most thermally stable (Fig. 6B,C). Remarkably, different amides show CSPs at high and low temperatures, indicating disparate structural sensitivities to cold and heat. Changes in peak intensity can be used to follow protein unfolding by NMR [47]; thus, we chose two well‐isolated amide peaks (N684 and S649) and extracted peak intensity as a function of temperature. To normalize for the change in intensity due to changing tumbling rates at different temperatures, we divided the intensities for N684 and S649 by the intensity of the most C‐terminal amide, which is unstructured at all temperatures. We found that N684 and S649 show maximum normalized peak intensities that coincide with the most folded point in thermal melts monitored by far‐UV circular dichroism (CD) spectroscopy (Fig. 6C,D). Collectively, these data demonstrate that apo LETM1 F‐EF senses cold and hot temperatures through unfolding of distinct regions of the domain.

### Isolated LETM1 F‐EF weakly and non‐selectively interacts with the LETM1 NTD and CC3 as well as the GHITM NTD and CTDs *in vitro*

**Fig. 7:**
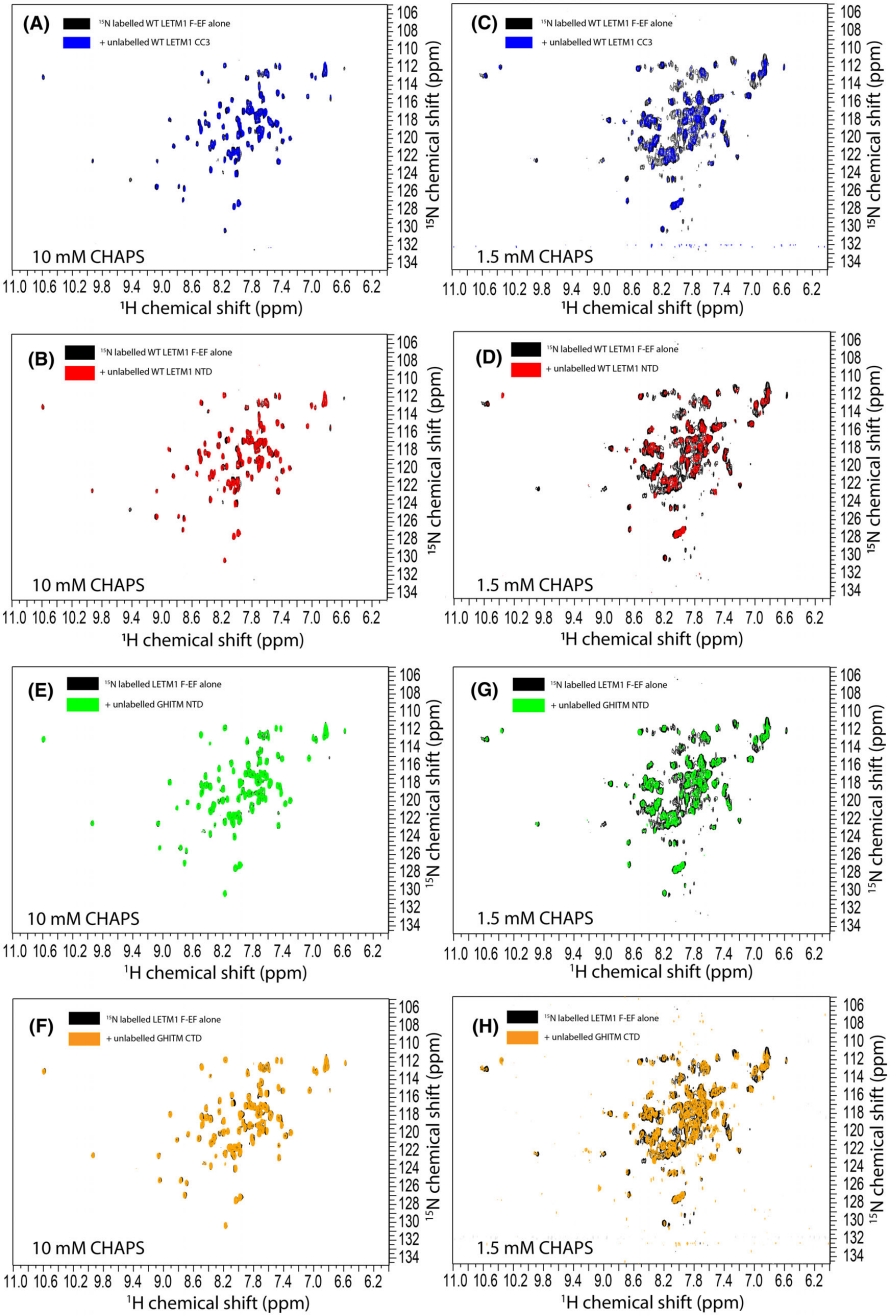
LETM1 F‐EF interactions with other LETM1 and GHITM domains. Overlaid ^1^H‐^15^N HSQC spectra of uniformly ^15^N‐labeled holo WT LETM1 F‐EF alone (black) and mixed with purified unlabeled (A) LETM1‐CC3 (blue) or (B) LETM1‐NTD (red) in the presence of 10 mm CHAPS. Overlaid ^1^H‐^15^N HSQC spectra of uniformly ^15^N‐labeled holo WT LETM1 F‐EF alone (black) and mixed with purified unlabeled (C) LETM1‐CC3 (blue) or (D) LETM1‐NTD in the presence of 1.5 mm CHAPS. Overlaid ^1^H‐^15^N HSQC spectra of uniformly ^15^N‐labeled holo WT LETM1 F‐EF alone (black) and mixed with purified unlabeled (E) GHITM‐NTD (green) or (F) GHITM‐CTD (orange) in the presence of 10 mm CHAPS. Overlaid ^1^H‐^15^N HSQC spectra of uniformly ^15^N‐labeled holo WT LETM1 F‐EF alone (black) and mixed with purified unlabeled (G) GHITM‐NTD (green) or (H) GHITM‐CTD (orange) in the presence of 1.5 mm CHAPS. In A, B, E and F, LETM1 F‐EF was ~200 μm while LETM1‐CC3, LETM1‐NTD, GHITM‐NTD and GHITM‐CTD was ~600, 600, 600 and 445 μm, respectively. In C, D, G and H, LETM1 F‐EF was ~110 μm while LETM1‐CC3, LETM1‐NTD, GHITM‐NTD and GHITM‐CTD was ~325, 325, 325 and 230 μm, respectively. All experiments were performed in 20 mm Tris, pH 7.8, 50 mm NaCl at 35 °C.

The hydrophobic cleft of Ca^2+^‐loaded EF‐hand sensors is the site of target protein binding [12, 14, 15]. We postulated that, outside the homodimerization with another LETM1 F‐EF domain we previously identified [46], the cleft may interact with other domains or proteins. For the case of intra/inter‐LETM1 protomer binding we explored two candidates: (a) the LETM1 CC3 that AlphaFold predicts interacts with F‐EF with a mean predicted aligned error (PAE) of 11.0 Å across all residues making up the domains [39] (Fig. S4A), and (b) the LETM1 N‐terminal domain (NTD) that is predicted to interact with Ca^2+^‐CaM based on the Calmodulin Target Database [48]. The bioinformatic tool specifically identifies LETM1 residues ~182–205 as a potential binding site, which would be located in the matrix based on the two TM LETM1 topology (Fig. 1A). Further, AlphaFold predicts F‐EF interacts with LETM1‐NTD with a mean PAE of 19.1 Å (Fig. S4B). To experimentally test these scenarios, we probed for an interaction between recombinantly expressed LETM1‐NTD (residues 115–208) or CC3 (residues 527–634) and uniformly ^15^N‐labeled WT LETM1 F‐EF‐hand. We found only minor CSPs and some decreased peak intensities in the Ca^2+^‐loaded WT LETM1 F‐EF spectrum when mixed with either excess CC3 or NTD (Fig. 7A,B). To rule out that the 10 mm CHAPS in our system mitigates stable interactions between the domains, we also acquired the mixed spectra with reduced 1.5 mm CHAPS, again finding minimal CSPs but greater losses in peak intensity in both the NTD and CC3 mixtures (Fig. 7C,D). Note that CHAPS is added to eliminate peak doubling in the F‐EF spectra [20, 49].

Recent studies suggest that LETM1 is a regulator of mitochondrial Ca^2+^/H^+^ exchange rather than directly functioning as a Ca^2+^/H^+^ exchanger; rather, GHITM is suggested to be the mitochondrial Ca^2+^/H^+^ antiporter that is regulated by LETM1 interactions [21]. GHITM (UniProt: Q9H3K2) is a seven TM protein residing in the IMM with a matrix facing NTD (residues 46–82) and IMS facing C‐terminal domain (CTD; residues 293–345). Furthermore, both the NTD and CTD of GHITM contain putative Ca^2+^‐CaM binding sites that may be recognized by holo LETM1 F‐EF given the homology to Ca^2+^‐loaded N‐CaM [20]. To test whether LETM1 F‐EF interacts with GHITM, constructs spanning the NTDs and CTDs of GHITM (*i.e*., residue ranges 46–82 and 293–345, respectively) were engineered and protein samples purified. AlphaFold predicts interactions between LETM1 F‐EF and the GHITM‐NTD and ‐CTD with mean PAE scores of 10.4 and 14.8 Å, respectively (Fig. S4C,D). To experimentally test for the interaction, we mixed excess GHITM‐NTD and ‐CTD proteins with ^15^N‐labeled WT LETM1 F‐EF and acquired a ^1^H‐^15^N HSQC spectra. Small CSPs were observed primarily for the GHITM‐CTD mixed sample (Fig. 7E,F). We repeated the experiments with reduced 1.5 mm CHAPS, observing mostly peak broadening for both mixed samples (Fig. 7G,H).

Collectively, the NMR data provides evidence for weak and non‐selective interactions between LETM1 F‐EF and other LETM1 domains as well as the GHITM‐NTD and ‐CTD (Fig. S5).

## Discussion

In our previous study, we elucidated the Ca^2+^‐loaded (*i.e*., holo) WT LETM1 EF‐hand domain structure, discovering that a complete sequence identifiable helix–loop–helix motif (*i.e*., E_1_‐loop3‐F_1_) was paired with a partial loop–helix (*i.e*., loop1‐F_0_) to form the LETM1 F_0_‐E_1_F_1_‐hand domain (9BA1; [20]). Here, we used the Ca^2+^ binding‐deficient and Ca^2+^‐insensitive D676A/N678A LETM1 F‐EF mutant to elucidate the apo structure. Notably, while the D676A/N678A LETM1 F‐EF mutant provides higher quality NMR spectra (Fig. 2A), the protein shares a highly similar ^1^H‐^15^N‐HSQC spectral fingerprint as apo WT LETM1 F‐EF, making it the ideal surrogate.

In sensory EF‐hand domains, Ca^2+^ binding commonly initiates a change from a closed to open state that exposes a hydrophobic pocket for target protein interactions [12, 14, 40, 50, 51, 52]. Holo LETM1 F‐EF is no exception, despite missing one of four helices that typically constitute an EF‐hand pair and domain (Fig. 2B). In fact, holo LETM1 F‐EF shows >30% more solvent exposed hydrophobic surface area than archetypal holo N‐CaM even with remarkably similar semi‐perpendicular interhelical angles (Table 2). The apo D676A/N678A LETM1 F‐EF mutant structure presented here reveals the mechanism by which this exposed hydrophobicity is suppressed. Rather than a closure of the E_1_ and F_1_ interhelical angle (θ) to a semi‐parallel orientation, apo LETM1 F‐EF maintains a semi‐perpendicular θ but shows a much greater ϕ angle that (a) leaves the F_1_‐helix C‐terminus ~5.7 Å closer to the E_1_‐helix, (b) packs F_1_ against the F_0_/E_1_ helix pair and (c) closes the structure, reducing the solvent exposed hydrophobic surface area by ~26% (Figs 3, 4, Table 2).

The most common structural homolog to the apo LETM1 F‐EF among experimentally determined protein structures is the CTD (or domain III) of RuvA. The RuvA CTD/domain III primarily mediates protein–protein interactions [53, 54], and congruently, we found apo LETM1 F‐EF maintains a high level of solvent exposed hydrophobicity (*i.e*., on par with holo N‐CaM; Table 2). Thus, LETM1 F‐EF may bind to different targets in the apo versus holo state as observed for CaM [55, 56]. The IQ motif can bind to CaM with differing affinities depending on the absolute sequence of the motif and the presence or absence of Ca^2+^, although the IQ motif binding is more typically associated with apo CaM interactions [55]. We used the Calmodulin Target Database [48] to identify putative apo (*i.e*., IQ motif) and holo (*i.e*., 1–10, 1–12 and 1–14 motifs) CaM interaction sites within LETM1 and within GHITM. We targeted these specific proteins since intramolecular LETM1 interactions (*i.e*., F‐EF:CC3) were predicted by AlphaFold and GHITM was identified as a top LETM1 interactor in a mass spectrometry study [21]. Only holo CaM binding sites were predicted within the sequences of LETM1 and GHITM by the Calmodulin Target Database tool. Thus, we tested for these interactions in the presence of Ca^2+^, finding that GHITM‐NTD and ‐CTD weakly bind to holo LETM1 F‐EF. However, weak interactions were also observed between LETM1 F‐EF and the LETM1‐NTD and ‐CC3 (Fig. 7), suggesting the high levels of solvent exposed hydrophobic surface area may facilitate promiscuous binding to multiple partners. However, we note that our interaction experiments were performed using recombinant fragments *in vitro*, and thus, validation in cells and tissues is required to support physiological relevance. Further, it is more likely that specific LETM1 F‐EF protein binding partners remain to be identified for both holo and apo states.

LETM1 F‐EF is sensitive to Ca^2+^, pH and temperature [46]. The tripartite sensitivity is not mutually exclusive, as low pH and low temperature increase and decrease Ca^2+^ binding affinity, respectively [46]. Here, we found an increase in the number of visible amide peaks at more acidic pH (Fig. 6). The higher H^+^ concentration outcompeting amide deuteration and increased α‐helicity for LETM1 F‐EF at lower pH [46] likely contributed to the increased number of visible peaks in the ^1^H‐^15^N‐HSQC spectra. Tracking the CSPs of neighboring I663 and I648 residues (*i.e*., in sequence and 3D space, respectively) of the apo mutant revealed an imidazole H662 pKa of 6.58, in‐line with the previously determined pKa of 6.39 for H662 of apo WT LETM1 F‐EF. The small discrepancy could be due to the D676A/N678A loop3 mutations. In fact, despite H662 being located within loop2 between the F_0_ preceding and E_1_ entering helices, we previously found that mutations in the distant Ca^2+^ binding loop3 cause large perturbations in the H662 chemical shifts [20]. Thus, H662 protonation likely impacts the binding loop3 allosterically through a similar network of interactions, resulting in the higher Ca^2+^ affinity we previously observed at low pH [46]. Nevertheless, we cannot rule out that imprecise solution pH measurements could have contributed to some or all the ~0.19 H662 pKa difference between apo WT and the double mutant studied here.

The collapse of amide crosspeaks toward the central region of the ^1^H‐^15^N HSQC spectra at 5 °C (*i.e*., low temperature) and 50 °C (*i.e*., high temperature) reinforce past data showing LETM1 F‐EF undergoes heat‐ and cold‐induced unfolding at temperatures above 0 °C [46]. A plot of the normalized amide peak intensities for S649 and N684 versus temperature, which could be clearly tracked over all temperatures, revealed maximal intensities at temperatures that corresponded to the most folded points in thermal melts acquired by CD spectroscopy (Fig. 6C,D). Further, the data demonstrate that LETM1 F‐EF undergoes cold‐unfolding at near physiological temperatures, thus endowing the domain with a physiologically relevant temperature sensing function. LETM1 F‐EF is a member of a small group of proteins that include the mitochondrial iron–sulfur transferase apo IscU and bacterial Yfh1, whose cold unfolding can be observed above 0 °C [57, 58, 59]. Similar to the LETM1 F‐EF, the cold unfolding of Yfh1 shows a regiospecific denaturing fingerprints dependent on hot and cold temperatures [60]. In both IscU and Yfh1, acidic residues are found in close proximity to the hydrophobic core where mutual repulsion may permit water to penetrate the core, leading to cold unfolding [59]. Intriguingly, LETM1 F‐EF contains a highly acidic exiting F_1_‐helix that may act as a similar electrostatic gate at low temperature.

In conclusion, the apo LETM1 F‐EF mutant structure complements the recently determined holo LETM1 F‐EF structure [20], together demonstrating that the domain functions as a Ca^2+^ sensor by undergoing a structural change from an open, highly hydrophobic holo state to a closed, less hydrophobic apo state, involving a ϕ pivot of the F_1_ helix rather than a decrease of the E_1_‐F_1_ θ interhelical angle (Movie S1). Further, we find that the holo LETM1 F‐EF interacts weakly and promiscuously with other LETM1 domains as well as with the GHITM‐NTD and ‐CTD. Further, solution NMR demonstrates hot versus cold‐regiospecific unfolding above 0 °C and a H662 pKa of ~6.58. The structural changes in response to Ca^2+^ and effects of physiological‐relevant changes in pH and temperature on Ca^2+^ binding affinity highlight LETM1 F‐EF as an adaptive regulatory element with tripartite sensitivity in the complex environment of the mitochondrial matrix.

## Author contributions

Q‐TL: conceptualization, methodology, formal analysis, investigation, writing – original draft, review and editing, visualization. DMC: methodology, formal analysis, investigation, revisions. PBS: conceptualization, writing – review and editing, resources, supervision, project administration, funding acquisition.

### Peer review

The peer review history for this article is available at https://www.webofscience.com/api/gateway/wos/peer‐review/10.1002/1873‐3468.70006.

## Supporting information


**Fig. S1.** Ramachandran plot of the apo D676A/N678A LETM1 F‐EF‐hand domain structure.
**Fig. S2.** ^1^H‐^15^N‐HSQCs of apo D676A/N678A LETM1 F‐EF in the absence and presence of EGTA and Ca^2+^ binding predictions by AlphaFold3.
**Fig. S3.** MD simulations (500 ns) of the experimentally resolved D676A/N678A (AEA) and homology modeled WT, D676K/N678K (KEK) and D676S/N678S (SES) LETM1 F‐EF structures.
**Fig. S4.** Alphafold LETM1 F‐EF domain interaction predictions.
**Fig. S5.** ^1^H‐^15^N‐HSQC ^15^N‐LETM1 F‐EF chemical shift perturbations (CSPs) induced by LETM1‐CC3, LETM1‐NTD, GHITM‐NTD and GHITM‐CTD domains.

**Movie S1.** LETM1 F‐EF‐hand domain Ca^2+^‐free (apo) to Ca^2+^‐bound (holo) conformational transition.

## Data Availability

Chemical shift assignments and atomic coordinates have been deposited in BioMagResBank (accession code 31205) and PDB (accession code 9DMR), respectively. The MD trajectory data generated in this study are freely available from zenodo.org under the accession links 10.5281/zenodo.X, where X is 14 541 820, 14 541 848, 14 541 873 and 14 541 901, to access the four different GROMACS 2022.3 trajectory (xtc) and energy (edr) files (500 ns each). All other data available upon request to PBS (pstatho@uwo.ca).

## References

[1] Endele S , Fuhry M , Pak SJ , Zabel BU and Winterpacht A (1999) LETM1, a novel gene encoding a putative EF‐hand Ca(2+)‐binding protein, flanks the wolf‐Hirschhorn syndrome (WHS) critical region and is deleted in most WHS patients. Genomics 60, 218–225. 10486213 10.1006/geno.1999.5881

[2] Schlickum S , Moghekar A , Simpson JC , Steglich C , O'Brien RJ , Winterpacht A and Endele SU (2004) LETM1, a gene deleted in wolf‐Hirschhorn syndrome, encodes an evolutionarily conserved mitochondrial protein. Genomics 83, 254–261. 14706454 10.1016/j.ygeno.2003.08.013

[3] Nowikovsky K , Froschauer EM , Zsurka G , Samaj J , Reipert S , Kolisek M , Wiesenberger G and Schweyen RJ (2004) The LETM1/YOL027 gene family encodes a factor of the mitochondrial K+ homeostasis with a potential role in the wolf‐Hirschhorn syndrome. J Biol Chem 279, 30307. 15138253 10.1074/jbc.M403607200

[4] Nowikovsky K , Reipert S , Devenish RJ and Schweyen RJ (2007) Mdm38 protein depletion causes loss of mitochondrial K+/H+ exchange activity, osmotic swelling and mitophagy. Cell Death Differ 14, 1647–1656. 17541427 10.1038/sj.cdd.4402167

[5] Austin S , Tavakoli M , Pfeiffer C , Seifert J , Mattarei A , De Stefani D , Zoratti M and Nowikovsky K (2017) LETM1‐mediated K(+) and Na(+) homeostasis regulates mitochondrial Ca(2+) efflux. Front Physiol 8, 839. 29204122 10.3389/fphys.2017.00839 PMC5698270

[6] Nakamura S , Matsui A , Akabane S , Tamura Y , Hatano A , Miyano Y , Omote H , Kajikawa M , Maenaka K , Moriyama Y *et al*. (2020) The mitochondrial inner membrane protein LETM1 modulates cristae organization through its LETM domain. Commun Biol 3, 99. 32139798 10.1038/s42003-020-0832-5 PMC7058069

[7] Aral C , Demirkesen S , Bircan R and Yasar Sirin D (2020) Melatonin reverses the oxidative stress and mitochondrial dysfunction caused by LETM1 silencing. Cell Biol Int 44, 795–807. 31777134 10.1002/cbin.11274

[8] Lin QT and Stathopulos PB (2019) Molecular mechanisms of leucine zipper EF‐hand containing transmembrane Protein‐1 function in health and disease. Int J Mol Sci 20, 286. 30642051 10.3390/ijms20020286 PMC6358941

[9] Tsai MF , Jiang D , Zhao L , Clapham D and Miller C (2014) Functional reconstitution of the mitochondrial Ca^2+^/H+ antiporter Letm1. J Gen Physiol 143, 67–73. 24344246 10.1085/jgp.201311096 PMC3874562

[10] Shao J , Fu Z , Ji Y , Guan X , Guo S , Ding Z , Yang X , Cong Y and Shen Y (2016) Leucine zipper‐EF‐hand containing transmembrane protein 1 (LETM1) forms a Ca(2+)/H(+) antiporter. Sci Rep 6, 34174. 27669901 10.1038/srep34174 PMC5037442

[11] Lee SY , Kang MG , Shin S , Kwak C , Kwon T , Seo JK , Kim JS and Rhee HW (2017) Architecture mapping of the inner mitochondrial membrane proteome by chemical tools in live cells. J Am Chem Soc 139, 3651–3662. 28156110 10.1021/jacs.6b10418

[12] Ikura M and Ames JB (2006) Genetic polymorphism and protein conformational plasticity in the calmodulin superfamily: two ways to promote multifunctionality. Proc Natl Acad Sci U S A 103, 1159–1164. 16432210 10.1073/pnas.0508640103 PMC1360552

[13] Chazin WJ (2011) Relating form and function of EF‐hand calcium binding proteins. Acc Chem Res 44, 171–179. 21314091 10.1021/ar100110d PMC3059389

[14] Grabarek Z (2006) Structural basis for diversity of the EF‐hand calcium‐binding proteins. J Mol Biol 359, 509–525. 16678204 10.1016/j.jmb.2006.03.066

[15] Ishida H and Vogel HJ (2013) EF‐hand proteins. In Encyclopedia of Metalloproteins ( Kretsinger RH , Uversky VN and Permyakov EA , eds), pp. 766–775. Springer New York, New York, NY.

[16] Kawasaki H and Kretsinger RH (2014) Structural differences among subfamilies of EF‐hand proteins – a view from the pseudo two‐fold symmetry axis. Proteins 82, 2915–2924. 24638959 10.1002/prot.24562

[17] Zhang M , Tanaka T and Ikura M (1995) Calcium‐induced conformational transition revealed by the solution structure of apo calmodulin. Nat Struct Mol Biol 2, 758–767. 10.1038/nsb0995-758 7552747

[18] Herzberg O and James MN (1988) Refined crystal structure of troponin C from Turkey skeletal muscle at 2.0 a resolution. J Mol Biol 203, 761–779. 3210231 10.1016/0022-2836(88)90208-2

[19] Maler L , Sastry M and Chazin WJ (2002) A structural basis for S100 protein specificity derived from comparative analysis of apo and Ca(2+)‐calcyclin. J Mol Biol 317, 279–290. 11902843 10.1006/jmbi.2002.5421

[20] Lin QT , Colussi DM , Lake T and Stathopulos PB (2024) An AI‐informed NMR structure reveals an extraordinary LETM1 F‐EF‐hand domain that functions as a two‐way regulator of mitochondrial calcium. Structure 32, 2063–2082.e5. 39317198 10.1016/j.str.2024.08.020

[21] Austin S , Mekis R , Mohammed SEM , Scalise M , Wang WA , Galluccio M , Pfeiffer C , Borovec T , Parapatics K , Vitko D *et al*. (2022) TMBIM5 is the Ca(2+)/H(+) antiporter of mammalian mitochondria. EMBO Rep 23, e54978. 36321428 10.15252/embr.202254978 PMC9724676

[22] Delaglio F , Grzesiek S , Vuister GW , Zhu G , Pfeifer J and Bax A (1995) NMRPipe: a multidimensional spectral processing system based on UNIX pipes. J Biomol NMR 6, 277–293. 8520220 10.1007/BF00197809

[23] Bartels C , Xia TH , Billeter M , Guntert P and Wuthrich K (1995) The program XEASY for computer‐supported NMR spectral analysis of biological macromolecules. J Biomol NMR 6, 1–10. 22911575 10.1007/BF00417486

[24] Shen, Y. & Bax, A. (2015) Protein structural information derived from NMR chemical shift with the neural network program TALOS‐N, Methods Mol Biol 1260, 17–32. 25502373 10.1007/978-1-4939-2239-0_2 PMC4319698

[25] Guntert P (2004) Automated NMR structure calculation with CYANA. Methods Mol Biol 278, 353–378. 15318003 10.1385/1-59259-809-9:353

[26] Wurz JM , Kazemi S , Schmidt E , Bagaria A and Guntert P (2017) NMR‐based automated protein structure determination. Arch Biochem Biophys 628, 24–32. 28263718 10.1016/j.abb.2017.02.011

[27] Nederveen AJ , Doreleijers JF , Vranken W , Miller Z , Spronk CA , Nabuurs SB , Guntert P , Livny M , Markley JL , Nilges M *et al*. (2005) RECOORD: a recalculated coordinate database of 500+ proteins from the PDB using restraints from the BioMagResBank. Proteins 59, 662–672. 15822098 10.1002/prot.20408

[28] Brunger AT , Adams PD , Clore GM , DeLano WL , Gros P , Grosse‐Kunstleve RW , Jiang JS , Kuszewski J , Nilges M , Pannu NS *et al*. (1998) Crystallography & NMR system: a new software suite for macromolecular structure determination. Acta Crystallogr D Biol Crystallogr 54, 905–921. 9757107 10.1107/s0907444998003254

[29] Holm L , Laiho A , Toronen P and Salgado M (2023) DALI shines a light on remote homologs: one hundred discoveries. Protein Sci 32, e4519. 36419248 10.1002/pro.4519 PMC9793968

[30] Yap KL , Ames JB , Swindells MB and Ikura M (2002) Vector geometry mapping. A method to characterize the conformation of helix‐loop‐helix calcium‐binding proteins. Methods Mol Biol 173, 317–324. 11859772 10.1385/1-59259-184-1:317

[31] Goddard TD , Huang CC , Meng EC , Pettersen EF , Couch GS , Morris JH and Ferrin TE (2018) UCSF ChimeraX: meeting modern challenges in visualization and analysis. Protein Sci 27, 14–25. 28710774 10.1002/pro.3235 PMC5734306

[32] Baryshnikova OK , Williams TC and Sykes BD (2008) Internal pH indicators for biomolecular NMR. J Biomol NMR 41, 5–7. 18398685 10.1007/s10858-008-9234-6

[33] Webb, B. & Sali, A. (2017) Protein structure modeling with MODELLER, Methods Mol Biol 1654, 39–54. 28986782 10.1007/978-1-4939-7231-9_4

[34] Abraham MJ , Murtola T , Schulz R , Páll S , Smith JC , Hess B and Lindahl E (2015) GROMACS: high performance molecular simulations through multi‐level parallelism from laptops to supercomputers. SoftwareX 1‐2, 19–25.

[35] Jorgensen WL , Maxwell DS and Tirado‐Rives J (1996) Development and testing of the OPLS all‐atom force field on conformational energetics and properties of organic liquids. J Am Chem Soc 118, 11225–11236.

[36] Berendsen HJC , Postma JPM , van Gunsteren WF , DiNola A and Haak JR (1984) Molecular dynamics with coupling to an external bath. J Chem Phys 81, 3684–3690.

[37] Parrinello M and Rahman A (1981) Polymorphic transitions in single crystals: a new molecular dynamics method. J Appl Phys 52, 7182–7190.

[38] Pettersen EF , Goddard TD , Huang CC , Meng EC , Couch GS , Croll TI , Morris JH and Ferrin TE (2021) UCSF ChimeraX: structure visualization for researchers, educators, and developers. Protein Sci 30, 70–82. 32881101 10.1002/pro.3943 PMC7737788

[39] Abramson J , Adler J , Dunger J , Evans R , Green T , Pritzel A , Ronneberger O , Willmore L , Ballard AJ , Bambrick J *et al*. (2024) Accurate structure prediction of biomolecular interactions with AlphaFold 3. Nature 630, 493–500. 38718835 10.1038/s41586-024-07487-w PMC11168924

[40] Herzberg O , Moult J and James MN (1986) A model for the Ca^2+^−induced conformational transition of troponin C. A trigger for muscle contraction. J Biol Chem 261, 2638–2644. 3949740

[41] Prabu JR , Thamotharan S , Khanduja JS , Chandra NR , Muniyappa K and Vijayan M (2009) Crystallographic and modelling studies on mycobacterium tuberculosis RuvA additional role of RuvB‐binding domain and inter species variability. Biochim Biophys Acta 1794, 1001–1009. 19374958 10.1016/j.bbapap.2009.04.003

[42] Varadi M , Anyango S , Deshpande M , Nair S , Natassia C , Yordanova G , Yuan D , Stroe O , Wood G , Laydon A *et al*. (2022) AlphaFold protein structure database: massively expanding the structural coverage of protein‐sequence space with high‐accuracy models. Nucleic Acids Res 50, D439–D444. 34791371 10.1093/nar/gkab1061 PMC8728224

[43] Wang L , Zhang M and Alexov E (2016) DelPhiPKa web server: predicting pKa of proteins, RNAs and DNAs. Bioinformatics 32, 614–615. 26515825 10.1093/bioinformatics/btv607 PMC5963359

[44] Webb H , Tynan‐Connolly BM , Lee GM , Farrell D , O'Meara F , Sondergaard CR , Teilum K , Hewage C , McIntosh LP and Nielsen JE (2011) Remeasuring HEWL pK(a) values by NMR spectroscopy: methods, analysis, accuracy, and implications for theoretical pK(a) calculations. Proteins 79, 685–702. 21287606 10.1002/prot.22886

[45] Santo‐Domingo J and Demaurex N (2012) Perspectives on: SGP symposium on mitochondrial physiology and medicine: the renaissance of mitochondrial pH. J Gen Physiol 139, 415–423. 22641636 10.1085/jgp.201110767 PMC3362525

[46] Lin QT , Lee R , Feng AL , Kim MS and Stathopulos PB (2021) The leucine zipper EF‐hand containing transmembrane protein‐1 EF‐hand is a tripartite calcium, temperature, and pH sensor. Protein Sci 30, 855–872. 33576522 10.1002/pro.4042 PMC7980503

[47] Pastore A , Martin SR , Politou A , Kondapalli KC , Stemmler T and Temussi PA (2007) Unbiased cold denaturation: low‐ and high‐temperature unfolding of yeast frataxin under physiological conditions. J Am Chem Soc 129, 5374–5375. 17411056 10.1021/ja0714538 PMC2664662

[48] Yap KL , Kim J , Truong K , Sherman M , Yuan T and Ikura M (2000) Calmodulin target database. J Struct Funct Genomics 1, 8–14. 12836676 10.1023/a:1011320027914

[49] Qin X , Liu M , Yang D and Zhang X (2010) Concentration‐dependent aggregation of CHAPS investigated by NMR spectroscopy. J Phys Chem B 114, 3863–3868. 20192181 10.1021/jp911720w

[50] Bhattacharya S , Bunick CG and Chazin WJ (2004) Target selectivity in EF‐hand calcium binding proteins. Biochim Biophys Acta 1742, 69–79. 15590057 10.1016/j.bbamcr.2004.09.002

[51] Lewit‐Bentley A and Rety S (2000) EF‐hand calcium‐binding proteins. Curr Opin Struct Biol 10, 637–643. 11114499 10.1016/s0959-440x(00)00142-1

[52] Marshall CB , Nishikawa T , Osawa M , Stathopulos PB and Ikura M (2015) Calmodulin and STIM proteins: two major calcium sensors in the cytoplasm and endoplasmic reticulum. Biochem Biophys Res Commun 460, 5–21. 25998729 10.1016/j.bbrc.2015.01.106

[53] Roe SM , Barlow T , Brown T , Oram M , Keeley A , Tsaneva IR and Pearl LH (1998) Crystal structure of an octameric RuvA‐Holliday junction complex. Mol Cell 2, 361–372. 9774974 10.1016/s1097-2765(00)80280-4

[54] Wyatt HD and West SC (2014) Holliday junction resolvases. Cold Spring Harb Perspect Biol 6, a023192. 25183833 10.1101/cshperspect.a023192 PMC4142969

[55] Andrews C , Xu Y , Kirberger M and Yang JJ (2020) Structural aspects and prediction of calmodulin‐binding proteins. Int J Mol Sci 22, 308. doi: 10.3390/ijms22010308 33396740 PMC7795363

[56] Tidow H and Nissen P (2013) Structural diversity of calmodulin binding to its target sites. FEBS J 280, 5551–5565. 23601118 10.1111/febs.12296

[57] Sanfelice D , Morandi E , Pastore A , Niccolai N and Temussi PA (2015) Cold denaturation unveiled: molecular mechanism of the asymmetric unfolding of yeast frataxin. ChemPhysChem 16, 3599–3602. 26426928 10.1002/cphc.201500765 PMC4676917

[58] Graziano G (2014) On the mechanism of cold denaturation. Phys Chem Chem Phys 16, 21755–21767. 25198426 10.1039/c4cp02729a

[59] Yan R , Rios PD , Pastore A and Temussi PA (2018) The cold denaturation of IscU highlights structure‐function dualism in marginally stable proteins. Commun Chem 1, 13. doi: 10.1038/s42004-018-0015-1 PMC7612454 35243006

[60] Puglisi R , Karunanithy G , Hansen DF , Pastore A and Temussi PA (2021) The anatomy of unfolding of Yfh1 is revealed by site‐specific fold stability analysis measured by 2D NMR spectroscopy. Commun Chem 4, 127. doi: 10.1038/s42004-021-00566-3 35243007 PMC7612453

[61] Crooks GE , Hon G , Chandonia JM and Brenner SE (2004) WebLogo: a sequence logo generator. Genome Res 14, 1188–1190. 15173120 10.1101/gr.849004 PMC419797

