# Supplementary material for: The apo LETM1 F‐EF‐hand adopts a closed conformation that underlies a multi‐modal sensory role in mitochondria
Source: FEBS Lett. 2025 Feb 10;599(7):971–88. doi: 10.1002/1873-3468.70006 (PMC11995678; doi:10.1002/1873-3468.70006)
Supplement: Supplementary file 1 — Fig. S1. Ramachandran plot of the apo D676A/N678A LETM1 F‐EF‐hand domain structure. Fig. S2. 1H‐15N‐HSQCs of apo D676A/N678A LETM1 F‐EF in the absence and presence of EGTA and Ca2+ binding predictions by AlphaFold3. Fig. S3. MD simulations (500 ns) of the experimentally resolved D676A/N678A (AEA) and homology modeled WT, D676K/N678K (KEK) and D676S/N678S (SES) LETM1 F‐EF structures. Fig. S4. Alphafold LETM1 F‐EF domain interaction predictions. Fig. S5. 1H‐15N‐HSQC 15N‐LETM1 F‐EF chemical shift perturbations (CSPs) induced by LETM1‐CC3, LETM1‐NTD, GHITM‐NTD and GHITM‐CTD domains. [file FEB2-599-971-s002.pdf]

PROCHECK-NMR

Ramachandran Plot
prchk_input (10 models)**

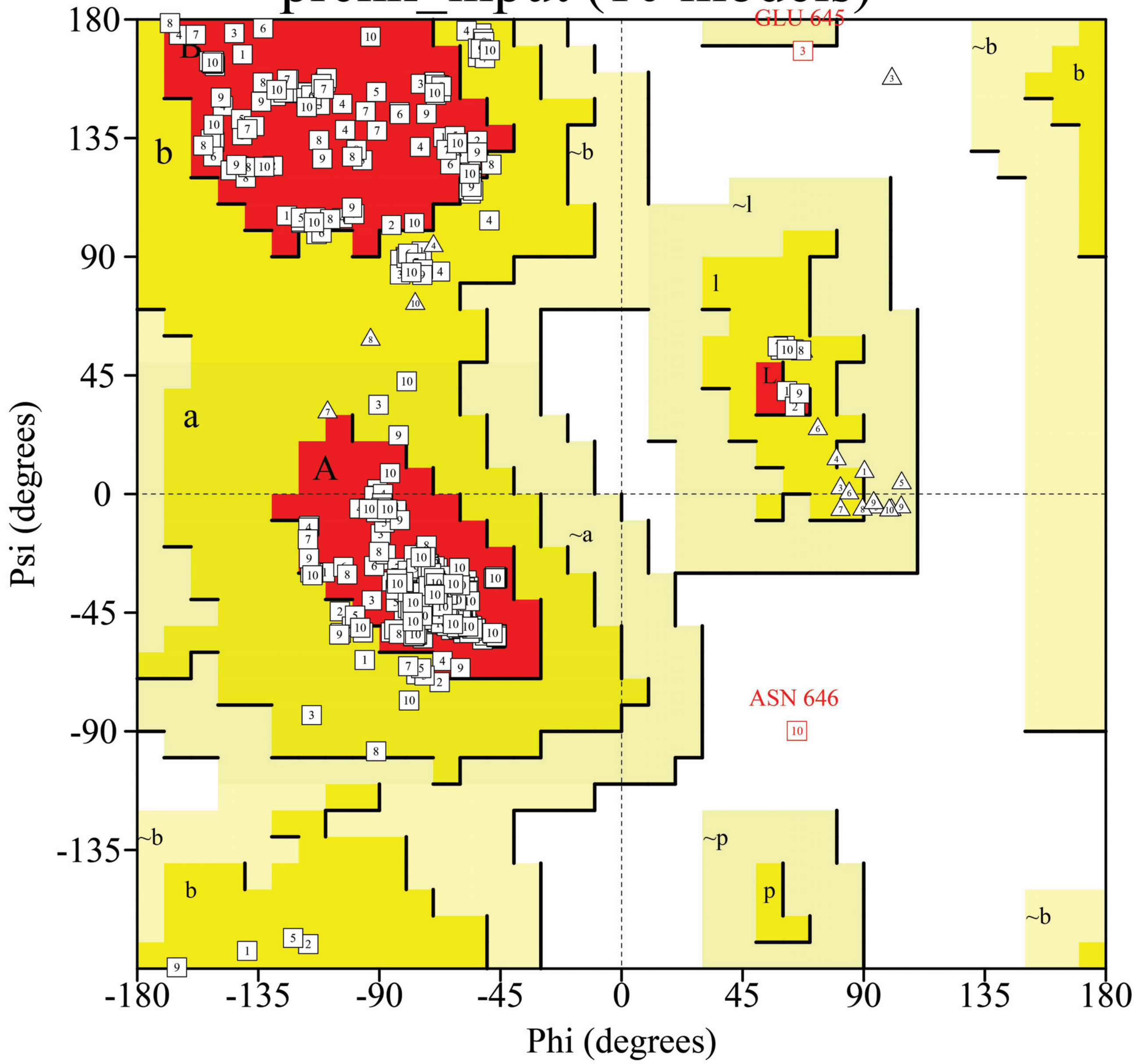

Plot statistics

Residues in most favoured regions [A,B,L]	469	88.5%
Residues in additional allowed regions [a,b,l,p]	59	11.1%
Residues in generously allowed regions [~a,~b,~l,~p]	0	.0%
Residues in disallowed regions	2	.4%
	----	------
Number of non-glycine and non-proline residues	530	100.0%
Number of end-residues (excl. Gly and Pro)	10	
Number of glycine residues (shown as triangles)	20	
Number of proline residues	10	

Total number of residues	570	

Based on an analysis of 118 structures of resolution of at least 2.0 Angstroms and R-factor no greater than 20%, a good quality model would be expected to have over 90% in the most favoured regions.
Model numbers shown inside each data point.

prchk_input_01.ps

** Selected residues only

Fig. S1

Figure S1. Ramachandran plot of the apo D676A/N678A LETM1 F-EF-hand domain structure. Ramachandran plot of the F-EF domain structure created by PROCHECK-NMR [24]. Shown are the dihedral ϕ and ψ angles for each residue of the 10 lowest energy structures. Two out of 530 non-Pro/non-Gly residues total in the 10 lowest energy structure ensemble were found in disallowed regions.

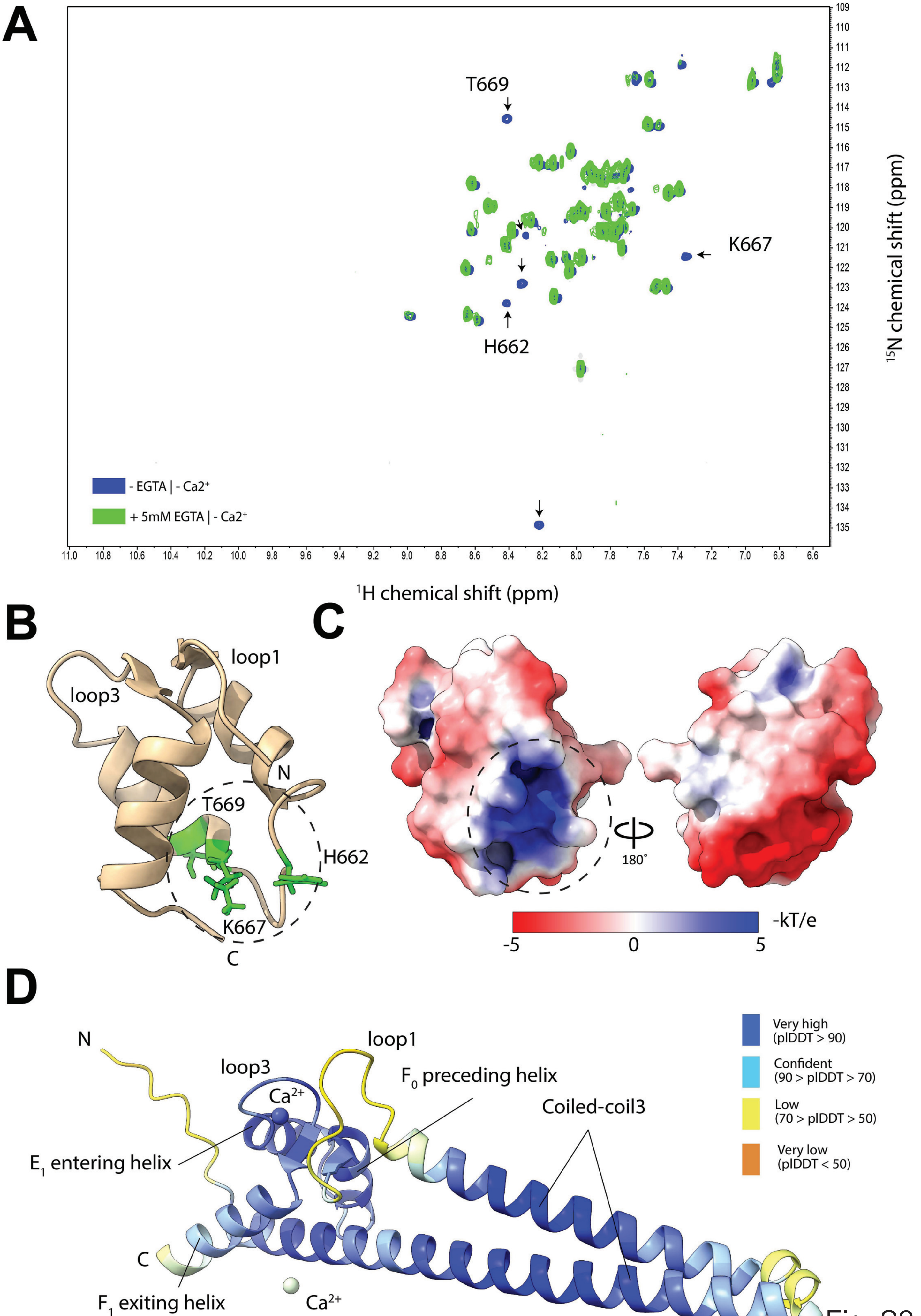
A
T669
K667
H662
- EGTA | - Ca2+
+ 5mM EGTA | - Ca2+
1H chemical shift (ppm)
15N chemical shift (ppm)
B
loop1
loop3
N
T669
H662
K667
C
C
180˚
-kT/e
-5
0
5
D
N
loop1
loop3
Ca2+
F0 preceding helix
Coiled-coil3
E1 entering helix
C
F1 exiting helix
Ca2+
Very high (plDDT > 90)
Confident (90 > plDDT > 70)
Low (70 > plDDT > 50)
Very low (plDDT < 50)

Fig. S2

Figure S2. ^{1}H-^{15}N-HSQCs of apo D676A/N678A LETM1 F-EF in the absence and presence of EGTA and Ca^{2+} binding predictions by AlphaFold3. (**A**) Overlaid ^{1}H-^{15}N HSQC spectra of uniformly ^{15}N-labeled apo D676A/N678A LETM1 F-EF in the absence (blue) and presence (green) of 5 mM EGTA. Peaks broadened by the addition of EGTA are indicated with black arrows. (**B**) Assigned residues (green sticks) identified in (*A*) to undergo broadening upon the addition of EGTA. (**C**) Electrostatic surface potential of apo D676A/N678A LETM1 F-EF calculated with the adaptive Poisson-Boltzmann solver (APBS). The gradient is from acidic (red) to basic (blue) potential in units of -kT/e, calculated at 37 °C, 0.15 M ionic strength, pH 7.8. (**D**) AlphaFold3 interaction predictions between human LETM1 F-EF with two Ca^{2+} ions. The structure and Ca^{2+} ions are colored based on pLDDT score (*i.e.* pLDDT > 90, dark blue; pLDDT between 70-90, light blue; pLDDT between 50-70, yellow; pLDDT < 50, orange). In (*B*-*C*), the dashed circles highlight the same regions on the cartoon and surface representations.

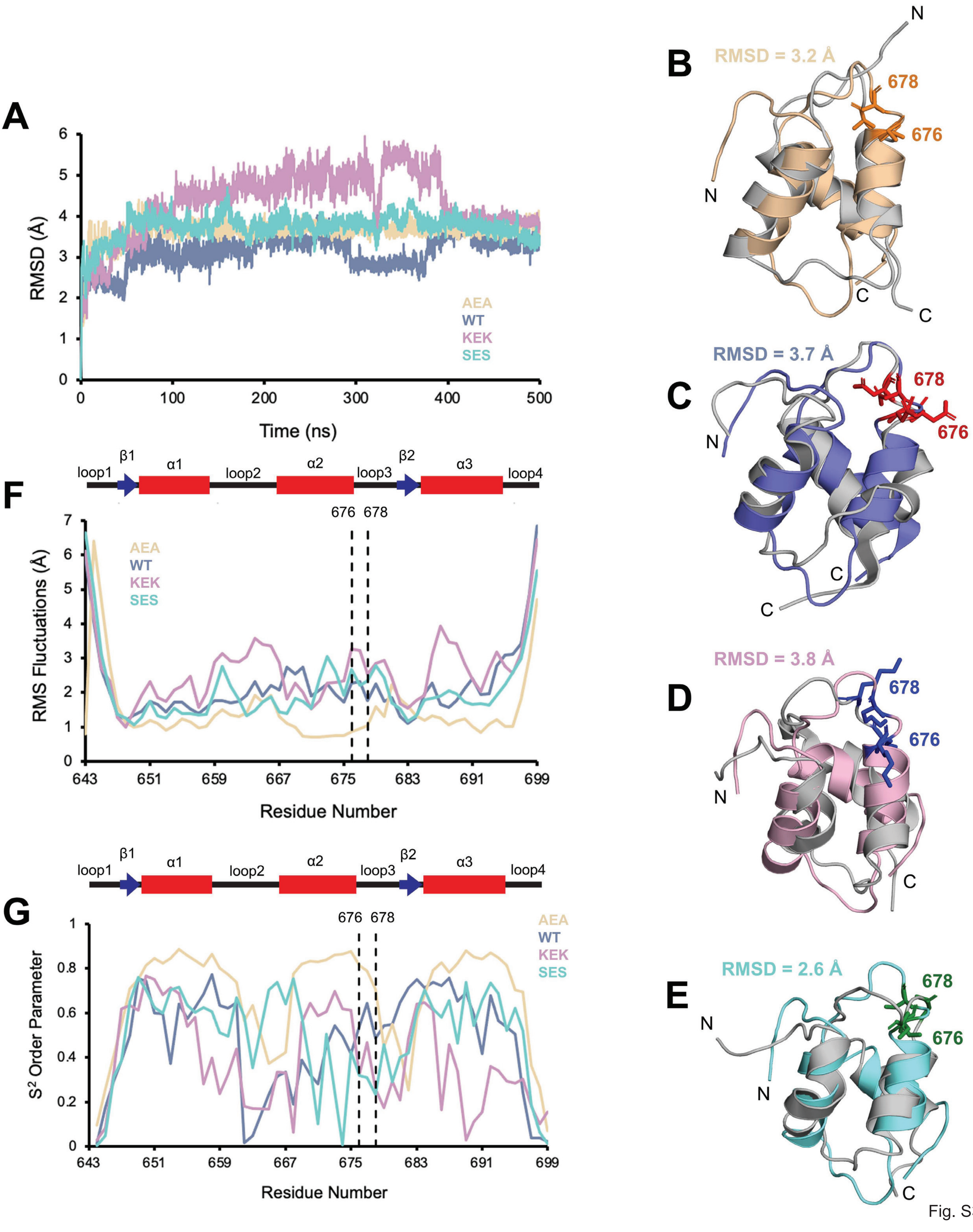
A
RMSD (Å)
Time (ns)
AEA
WT
KEK
SES
B
RMSD = 3.2 Å
678
676
N
C
C
RMSD = 3.7 Å
678
676
N
C
C
D
RMSD = 3.8 Å
678
676
N
C
E
RMSD = 2.6 Å
678
676
N
N
C
F
loop1
β1
α1
loop2
α2
loop3
β2
α3
loop4
676
678
RMS Fluctuations (Å)
AEA
WT
KEK
SES
Residue Number
G
loop1
β1
α1
loop2
α2
loop3
β2
α3
loop4
676
678
S² Order Parameter
AEA
WT
KEK
SES
Residue Number

Fig. S3

Figure S3. MD simulations (500 ns) of the experimentally resolved D676A/N678A (AEA) and homology modeled WT, D676K/N678K (KEK) and D676S/N678S (SES) LETM1 F-EF structures. (**A**) The Cα root-mean-square deviation (RMSD) of residues V647-V695 versus time, relative to the starting conformations. (**B**) Snapshots of the AEA LETM1 F-EF structure at 0 ns (beige) and 500 ns (grey). (**C**) Snapshots of the WT LETM1 F-EF homology model at 0 ns (purple) and 500 ns (grey). (**D**) Snapshots of the KEK LETM1 F-EF homology model at 0 ns (pink) and 500 ns (grey). (**E**) Snapshots of the SES LETM1 F-EF homology model at 0 ns (cyan) and 500 ns (grey). (**F**) Residue-specific order parameter (S^2), averaged over the total simulation time. (**G**) The residue-specific Cα root-mean-square fluctuation (RMSF), averaged over the total simulation time. In (*A*-*G*), AEA, WT, KEK and SES data are shown in beige, purple, pink and cyan, respectively. In (*B*-*E*), alignments were done using Cα atoms of the secondary structure elements only, the Cα RMSDs of the structure elements are indicated at top left and the 676 and 678 side chains are shown as sticks in each of the snapshots. In (*F*-*G*), the locations of the AEA secondary structure components relative to sequence space are shown at top.

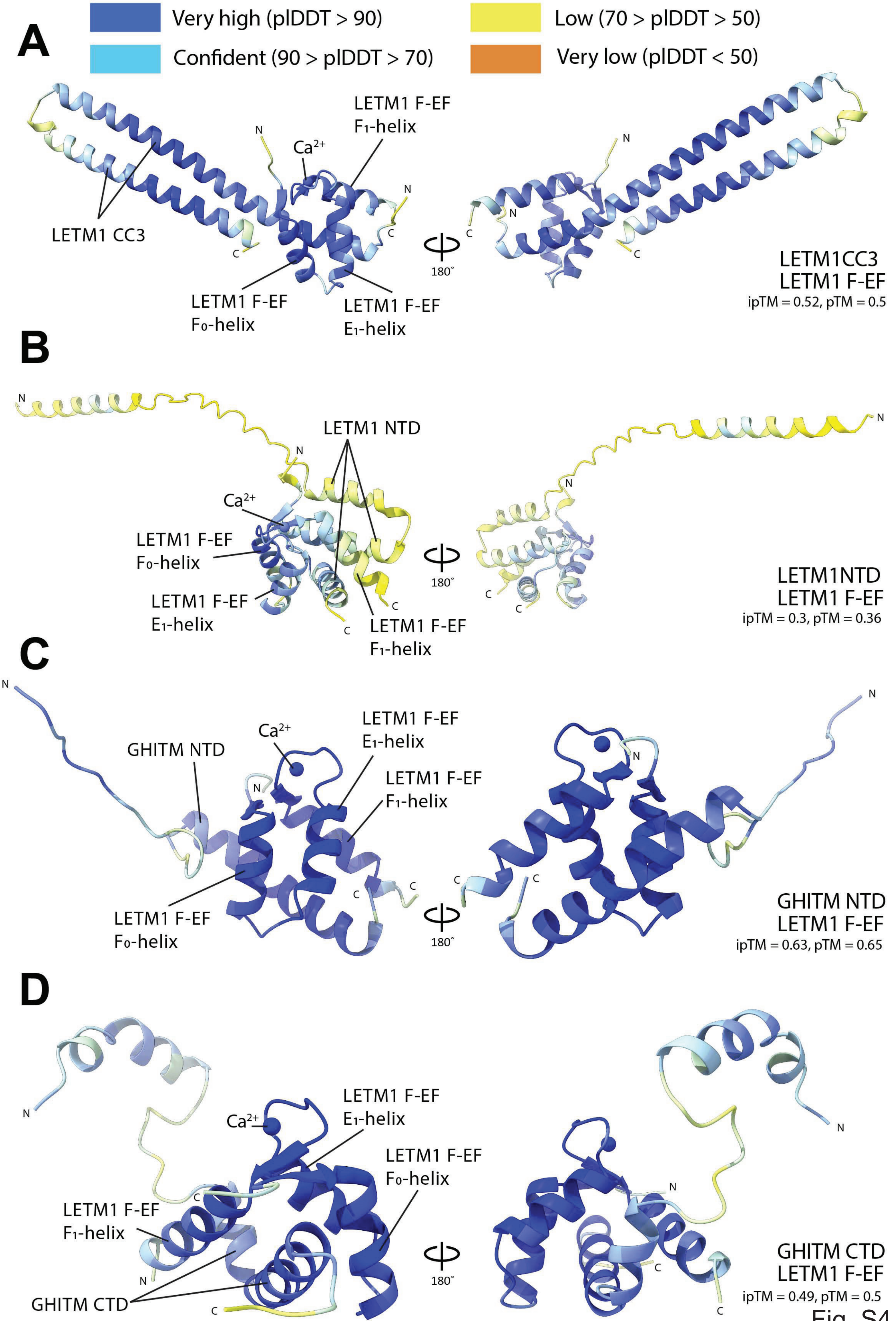
Very high (plDDT > 90)
Confident (90 > plDDT > 70)
Low (70 > plDDT > 50)
Very low (plDDT < 50)
A
LETM1 CC3
N
Ca2+
LETM1 F-EF
F1-helix
N
C
C
LETM1 F-EF
F0-helix
LETM1 F-EF
E1-helix
180˚
N
N
C
C
LETM1CC3
LETM1 F-EF
ipTM = 0.52, pTM = 0.5
B
N
LETM1 NTD
N
Ca2+
LETM1 F-EF
F0-helix
LETM1 F-EF
E1-helix
C
C
LETM1 F-EF
F1-helix
180˚
N
N
C
C
LETM1NTD
LETM1 F-EF
ipTM = 0.3, pTM = 0.36
C
N
GHITM NTD
Ca2+
LETM1 F-EF
E1-helix
LETM1 F-EF
F1-helix
N
C
C
LETM1 F-EF
F0-helix
180˚
N
N
C
C
GHITM NTD
LETM1 F-EF
ipTM = 0.63, pTM = 0.65
D
N
Ca2+
LETM1 F-EF
E1-helix
LETM1 F-EF
F0-helix
C
LETM1 F-EF
F1-helix
N
GHITM CTD
C
180˚
N
N
C
C
GHITM CTD
LETM1 F-EF
ipTM = 0.49, pTM = 0.5

Fig. S4

Figure S4. Alphafold LETM1 F-EF domain interaction predictions. Interaction predictions between human LETM1 F-EF in the presence of Ca^{2+} with (**A**) human LETM1-CC3, (**B**) human LETM1-NTD, (**C**) human GHITM-NTD and (**D**) human GHITM-CTD. In *A* – *D*, residue ranges for each interaction are the same as those used in our experimental assessments (**Fig. 7**), structures are coloured based on pLDDT score (*i.e.* pLDDT > 90, dark blue; pLDDT between 70-90, light blue; pLDDT between 50-70, yellow; pLDDT < 50, orange) and ipTM, pTM scores are labeled as output by AlphaFold3 [25].

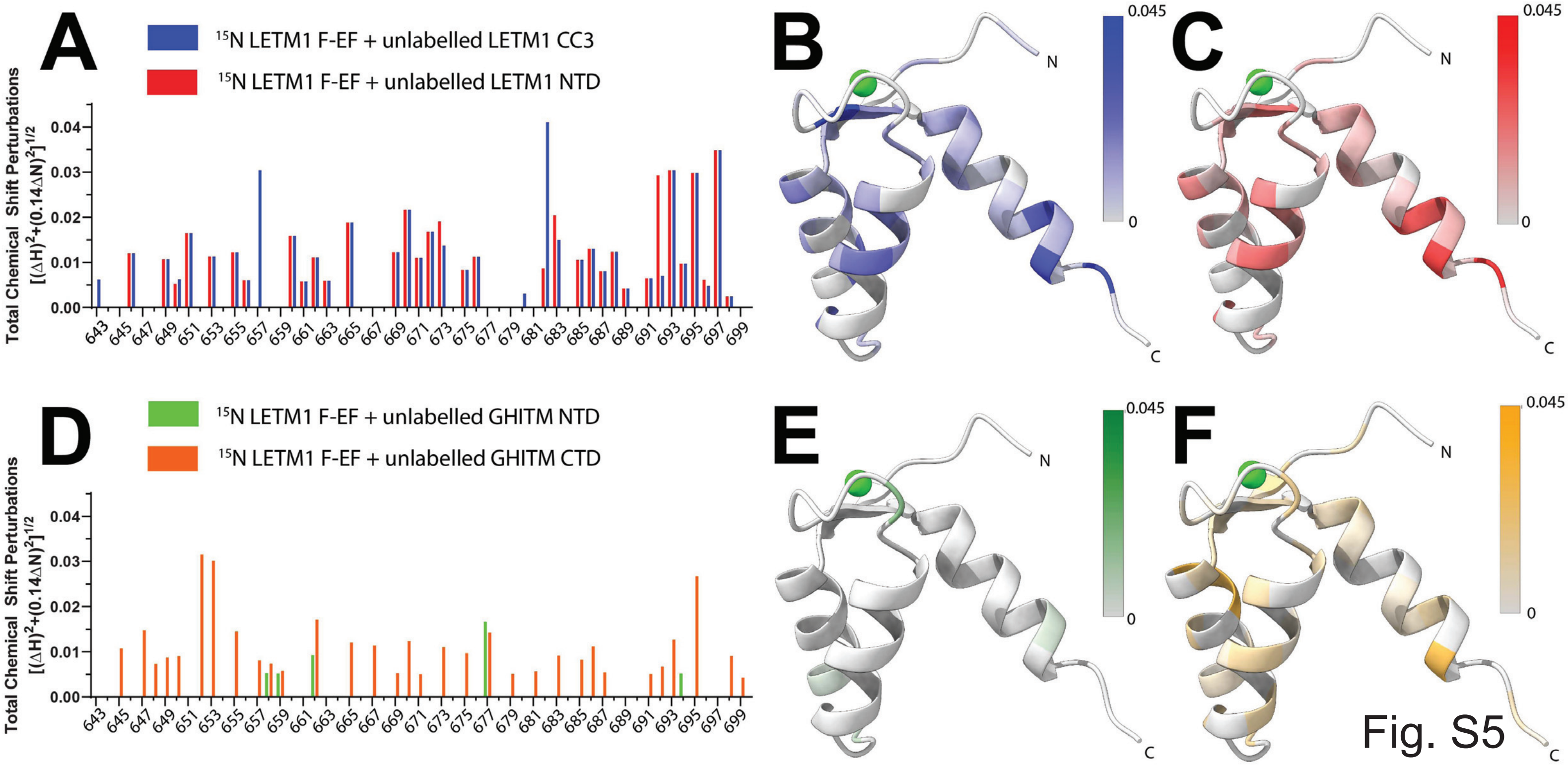
A
^{15}N LETM1 F-EF + unlabelled LETM1 CC3
^{15}N LETM1 F-EF + unlabelled LETM1 NTD
Total Chemical Shift Perturbations
$[(\Delta H)^2+(0.14\Delta N)^2]^{1/2}$
0.00
0.01
0.02
0.03
0.04
B
C
0.045
0
N
C
D
^{15}N LETM1 F-EF + unlabelled GHITM NTD
^{15}N LETM1 F-EF + unlabelled GHITM CTD
Total Chemical Shift Perturbations
$[(\Delta H)^2+(0.14\Delta N)^2]^{1/2}$
E
F

Fig. S5

Figure S5. ^{1}H-^{15}N-HSQC ^{15}N-LETM1 F-EF chemical shift perturbations (CSPs) induced by LETM1-CC3, LETM1-NTD, GHITM-NTD and GHITM-CTD domains. (**A**) Normalized total ^{1}H(^{15}N) CSPs caused by mixing unlabeled LETM1-CC3 (blue) or LETM1-NTD (red) with ^{15}N-LETM1 F-EF. (**B**) LETM1-CC3-induced total CSPs plotted on the holo LETM1 F-EF structure as a gradient from 0 (white) to 0.045 (blue). (**C**) LETM1-NTD-induced total CSPs plotted on the holo LETM1 F-EF structure as a gradient from 0 (white) to 0.045 (red). (**D**) Normalized total ^{1}H(^{15}N) CSPs caused by mixing unlabeled GHITM-NTD (green) or GHITM-CTD (orange) with ^{15}N-LETM1 F-EF. (**E**) GHITM-NTD-induced total CSPs plotted on the holo LETM1 F-EF structure as a gradient from 0 (white) to 0.045 (green). (**F**) GHITM-CTD-induced total CSPs plotted on the holo LETM1 F-EF structure as a gradient from 0 (white) to 0.045 (orange). In *A-F*, data are derived from ^{1}H-^{15}N-HSQCs shown in *Fig. 7A, E, C, G*. In *B, C, E and F* structural coordinates are 9BA1, and images were rendered in ChimeraX.
